# Modular Splicing Is Linked to Evolution in the Synapse-Specificity Molecule Kirrel3

**DOI:** 10.1523/ENEURO.0253-23.2023

**Published:** 2023-12-04

**Authors:** Dimitri Traenkner, Omar Shennib, Alyssa Johnson, Adam Weinbrom, Matthew R. Taylor, Megan E. Williams

**Affiliations:** Department of Neurobiology, University of Utah, School of Medicine, Salt Lake City, UT 84112

**Keywords:** alternative splicing, cell adhesion, evolution, Kirrel3, synapse

## Abstract

Kirrel3 is a cell-adhesion molecule that instructs the formation of specific synapses during brain development in mouse and Kirrel3 variants may be risk factors for autism and intellectual disabilities in humans. Kirrel3 is predicted to undergo alternative splicing but brain isoforms have not been studied. Here, we present the first in-depth characterization of Kirrel3 isoform diversity in brain using targeted, long-read mRNA sequencing of mouse hippocampus. We identified 19 isoforms with predicted transmembrane and secreted forms and show that even rare isoforms generate detectable protein in the brain. We also analyzed publicly-available long-read mRNA databases from human brain tissue and found 11 Kirrel3 isoforms that, similar to mouse, encode transmembrane and secreted forms. In mice and humans, Kirrel3 diversity arises from alternative, independent use of protein-domain coding exons and alternative early translation-stop signals. Intriguingly, the alternatively spliced exons appear at branch points in the chordate phylogenetic tree, including one exon only found in humans and their closest living relatives, the great apes. Together, these results validate a simple pipeline for analyzing isoform diversity in genes with low expression and suggest that Kirrel3 function is fine-tuned by alternative splicing and may play a role in brain evolution.

## Visual Abstract

**Figure:**
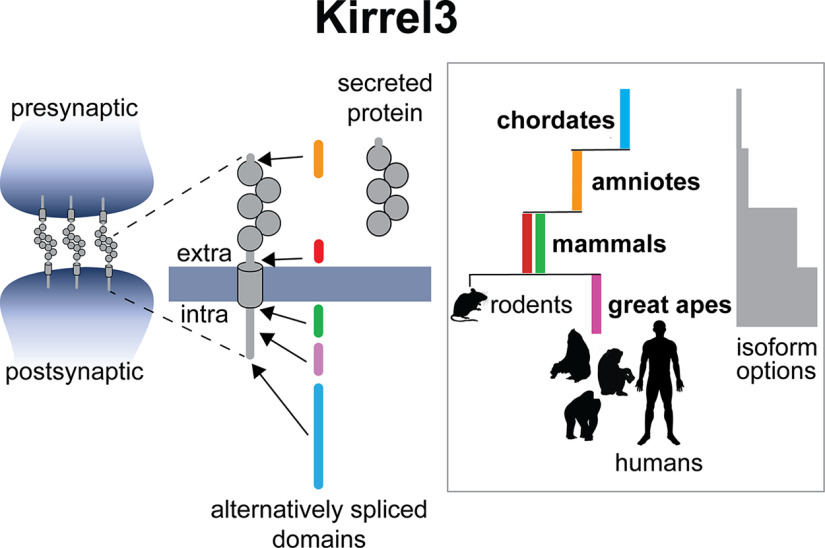

## Significance Statement

Kirrel3 is an important molecule for synapse and circuit formation with gene variants that are associated with neurodevelopmental disorders, yet Kirrel3 function remains largely unknown. Here, we report new isoforms of mouse and human Kirrel3, including secreted and transmembrane forms, that suggest a diverse repertoire of Kirrel3 actions. Importantly, we identified a new Kirrel3 exon only present in humans and the other great apes with potential to play an important role in circuit formation unique to these species.

## Introduction

Proper wiring of the mammalian brain requires billions of neurons to form synaptic connections with specific neurons. During development, axons extend to correct brain regions and then synapse with suitable neuronal partners, a process largely governed by the cellular environment, morphology, and cell surface proteins. Cell surface proteins help the cell find matching and avoid nonmatching synaptic partners (Sanes and Zipursky, 2020; Südhof, 2021). They also function in development by contributing to synapse formation and, likely, continue to function throughout adulthood during synapse maintenance, transmission, and plasticity.

The combinatorial expression of synaptic cell surface proteins provides different cell types with a unique identity that can be further tuned by alternative splicing. Alternative splicing of synaptic molecules can affect their protein-protein interactions and intracellular signaling (Südhof, 2017; Ovando-Zambrano et al., 2019; Li et al., 2020; Gomez et al., 2021; Trotter et al., 2023). This, in turn, expands the functional repertoire of synaptic genes and adds a layer of regulatory control. Accordingly, splicing factors are important for all aspects of neural development including neuronal health and accurate neuronal wiring (Furlanis and Scheiffele, 2018; Traunmüller et al., 2023). Despite the emerging importance and widespread use of alternative splicing programs in synaptic proteins (Ray et al., 2020), isoform diversity has been characterized for relatively few cell adhesion proteins including Dscam, Neurexin, and the Neurexin binding partners Neuroligin, Teneurin, and Latrophilin (Hattori et al., 2008; Schreiner et al., 2014; Treutlein et al., 2014; Südhof, 2017; Ovando-Zambrano et al., 2019; Li et al., 2020; Gomez et al., 2021).

Kirrel3 is a single pass transmembrane protein in the immunoglobulin (Ig) superfamily with five extracellular Ig domains and an intracellular PDZ-binding domain. Kirrel3 mediates cell-adhesion and synapse formation via trans-cellular, homophilic interactions and is essential for normal brain connectivity in mice (Prince et al., 2013; Martin et al., 2015, 2017; Roh et al., 2017; Brignall et al., 2018; Taylor et al., 2020; J. Wang et al., 2021). The Kirrel3 gene is composed of many small exons predicted to undergo alternative splicing. So far, three isoforms were reported in skeletal muscle (Durcan et al., 2014), but Kirrel3 isoform diversity has not been tested in the brain. Understanding the alternative splicing program of Kirrel3 is expected to provide new insight to its function in synapse and circuit formation. In addition, understanding the alternative splicing of Kirrel3 may facilitate the study of disease mechanisms that involve this synaptic gene because human Kirrel3 missense variants have been repeatedly identified as risk factors for neurodevelopmental disorders including autism spectrum disorders and intellectual disabilities (Bhalla et al., 2008; De Rubeis et al., 2014; Iossifov et al., 2014; T. Wang et al., 2016; Yuen et al., 2016; Li et al., 2017; Kalsner et al., 2018; Guo et al., 2019; Leblond et al., 2019; Hildebrand et al., 2020; Taylor et al., 2020; Zhou et al., 2022).

Here, we generated a comprehensive list of Kirrel3 isoforms expressed in the mouse hippocampus obtained with targeted long-read mRNA sequencing. In addition, we examined existing long-read transcriptome data from multiple mouse and human brain regions. We identified a total of 19 mouse and 11 human alternative transcripts predicted to encode distinct Kirrel3 proteins including secreted and transmembrane variants. In both mice and humans, Kirrel3 isoform diversity originates in the independent combination of four protein coding exons, two on the extracellular and two on the intracellular side, together with several alternative C termini. The four alternatively spliced protein coding exons first appear at different critical branching points in the chordate phylogenetic tree. Moreover, we identified a new alternatively spliced protein-coding Kirrel3 exon exclusively present in humans and the great apes (Hominidae), suggesting a key role of Kirrel3 in regulating brain connectivity in these closely related species.

## Materials and Methods

### Animals

Kirrel3 knock-out (KO) mice were described previously (Prince et al., 2013) and are backcrossed to the C57Bl/6J strain background. Male and female mice were used in equal numbers and described in the text. All animal experiments were approved and conducted in accordance with the University of Utah Institutional Animal Care and Use Committee (IACUC).

### Iso-seq sample processing and analysis

RNA was purified from two whole hippocampi per sample and a total of six samples. Samples are from two C57Bl/6J P14 males and females, respectively, as well as one P14 male and female knock-out control (KO; Prince et al., 2013). RNA was tested for quality (Agilent TapeStation, RIN ≥ 8.0) and converted to cDNA (NEBNext Single Cell/Low Input RNA Library Prep kit for Illumina). cDNA from each sample was amplified and barcoded in a mild stringency PCR (20 cycles of 30 s at 98°C, 54°C and 72°C), using a target-specific forward primer matching exon 2 of mouse Kirrel3 and a universal cDNA-reverse primer (Extended Data Table 1-1). Barcoded PCR products ≥1 kb were enriched (ProNex beads) and quantified (TapeStation D5000). Equal amounts of each sample were combined into a single 500 ng SMRTbell library for full-length transcript sequencing on a single SMRTcell using the Sequel II system (Pacific Bio). Iso-seq analysis was performed using R and the R-package BioStrings (Lifschitz et al., 2022). Results were manually confirmed and aligned using ApE plasmid editor software (Davis and Jorgensen, 2022). In brief, Iso-seq reads were demultiplexed using unique 16-bp bar code sequences and no more than two mismatches as search pattern. This strategy unambiguously identified the sample origin for 85.8% of all reads. Preliminary inspection of Iso-seq Kirrel3 transcripts was performed by dividing the Kirrel3 gene (NCBI Gene ID: 67703, 550,823 bp) into 155-bp fragments, and screening Iso-seq full-length reads for the presence of any of the 3536 resulting fragments. The preliminary inspection revealed one new exon and seven new exon extensions for a total of 29 alternatively spliced genomic segments (Extended Data Table 2-1). Iso-seq reads with sample IDs were screened for each of these 29 segments with no more than 40% mismatches.

### Sequence Read Archive (SRA) and phylogenetic analysis

Published mouse or human PacBio SMRT transcript libraries generated with brain derived tissues or cell lines were screened for Kirrel3 transcripts using the conserved exon 2 sequence and the NCBI blastn suite (Altschul et al., 1990). Similar to the analysis of mouse transcripts, human Kirrel3 transcripts were first inspected for the presence of any of 3772 elements, each 155 bp in length, that make up the human Kirrel3 gene (NCBI Gene ID: 84623, 584,513 bp). The preliminary inspection revealed three new exons and one new exon extension for a total of 27 alternatively spliced genomic segments (Extended Data Table 2-1). Similar to the Iso-seq analyses, mouse and human Kirrel3 transcripts were then examined for the presence of the respective alternatively spliced segments (≤40% mismatches) and manually aligned using BioStrings and ApE plasmid editor software, respectively. Phylogenetic analyses of mouse exons 6, 18, 19b, and 22 and human exons 3, 16, 17c, 19a, and 21 were performed by comparing nucleotide sequences to published genomes using the blastn suite (Altschul et al., 1990), direct gene ortholog inspection and by examining relationship of species with matches using the NCBI Taxonomy database (Schoch et al., 2020).

### Fluorescent *in situ* hybridization chain reaction (HCR)

HCR was performed as previously described (Trivedi et al., 2018). Mouse brains were cryo-sectioned to 30-μm slices, mounted on slides, fixed [4% paraformaldehyde (PFA)] and washed in PBS. Before processing samples according to protocol HCR v3.0 (Invitrogen), slices were treated with 1 μg/ml proteinase K-treated (TE buffer) and equilibrated in SSC buffer. Custom HCR probes were designed and generated by Molecular Instruments based on provided targets (Extended Data Table 4-1). After nuclear staining with Hoechst in PBS, coverslips were mounted in Fluoromount-G (Southern Biotech catalog #0100-01) and imaged (Zeiss LSM 710).

### Immunoblotting

Immunoblotting was conducted by standard techniques. Mouse samples are hippocampi from a three-week-old Kirrel3 wild-type or knock-out animals. Human samples are derived from deidentified postmortem dorsolateral prefrontal cortex stored at −80°C (kindly provided by Hilary Coon). Tissue samples are from one female (21 years, post mortem interval: 11.2 h) and one male (23 years, post mortem interval: 11.6 h). Mouse and human samples were processed in a Dounce homogenizer in lysis buffer (50 mm Tris, pH 7.5, 150 mm NaCl, 5 mm EDTA, 10% Glycerol, 1% CHAPS) with protease inhibitors at 100 mg/ml. The lysate was boiled in Laemmli buffer, run in a 10% bis-tris gel, and transferred to a nitrocellulose membrane. Antibodies used included mouse-anti-Pan-Kirrel3 (Neuromab, RRID: AB_2315857), mouse anti-Kirrel3^exon19b^ (Neuromab, RRID: AB_2341106), mouse-anti-GAPDH (Millipore, catalog #AB2302, RRID: AB_10615768), and a custom rabbit anti-Kirrel3 made against a C-terminal peptide (Martin et al., 2015). Secondary antibodies were goat-anti-mouse-HRP (Jackson ImmunoResearch, catalog #115-035-003, RRID: AB_10015289) and goat-anti-rabbit-HRP (Jackson ImmunoResearch, catalog #111-036-003, RRID: AB_2337942).

### DNA constructs

cDNAs and plasmids were generated using standard PCR-based restriction enzyme cloning (Taylor et al., 2020). Extracellular FLAG tags were added to mouse Kirrel3 Isoforms F (Kirrel3F) and K (Kirrel3K) in frame after the predicted signal-peptide cleavage site (exon 6) and the tagged constructs placed 3′ to mCherry or GFP followed by a viral 2A peptide sequence into the mammalian expression vector pBOS (mCherry-2A-FLAG-Kirrel3F/K).

### Cell aggregation assay

CHO cells were transfected with either mCherry-2A-FLAG-Kirrel3F, mCherry-2A-FLAG-Kirrel3K, GFP-2A-FLAG-Kirrel3F, or pBOS with mCherry only (mCherry-pBOS). After 48 h, transfected cells were washed and detached (0.01% trypsin) in magnesium-free HEPES buffer (HMF; 137 mm NaCl, 5.4 mm KCl, 1 mm CaCl2, 0.34 mm Na_2_HPO_4_, 5.6 mm glucose, and 10 mm HEPES, pH 7.4), spun down, and resuspended in HMF. Next, 100 000 cells suspended in 0.5 ml HMF were allowed to aggregate for 90 min (nutator, 37°C) in BSA-coated 24-well plates. For analysis, cells are fixed by adding paraformaldehyde (PFA; 4% final) and allowed to settle over a 24-h period. All but 0.3 ml of the supernatant are removed, cells and cell aggregates are carefully transferred in the remaining volume to a 96-well glass bottom plate, and the entire well is imaged (Zeiss LSM 710). Finally, the fraction of aggregated cells in the entire well was determined using ImageJ software.

### Immunocytochemistry and analysis

293T-HEK cells on poly-D-Lysine treated glass coverslips were transfected using PEI following a previously published protocol (Xie et al., 2013). After 24 h, cells were fixed (4% PFA) and rinsed with PBS and blocked for 30 min with 3% BSA/0.1% Triton X-100 in PBS (blocking buffer). Blocking buffer was also used for all subsequent washes and antibody dilutions. For antigen labeling, cells were incubated for 1–2 h with primary antibody at room temperature, washed three times, and incubated for 45 min with secondary antibody. After nuclear staining with Hoechst in PBS, coverslips were mounted in Fluoromount-G and imaged (Zeiss LSM 710). The relative enrichment of membrane proteins in cell-to-cell contacts was measured as ratio of average fluorescence pixel intensity along a cell’s contact versus free membrane using ImageJ software.

### Statistics

For the CHO aggregation experiment, the sample size (*n* = 3 for all conditions) indicates independent experiments conducted on different days. Groups were compared by a one-way ANOVA followed by pair-wise post-tests. For the cell junctional enrichment assay, we sampled several cells from three independent cultures and used a nested one-way ANOVA followed by pair-wise post-tests.

### Availability of data and materials

Iso-seq sequencing data are available at NCBI under SRA data file number PRJNA992104. Kirrel3 isoform sequence files have been deposited at NCBI. GenBank accession numbers for mouse Kirrel3 mRNA Isoforms F–T are OR239801–OR239815, respectively. All other materials and data that are not commercially available will be freely provided on reasonable request.

## Results

### A strategy to enrich for full-length Kirrel3 transcripts

**Figure 1.**
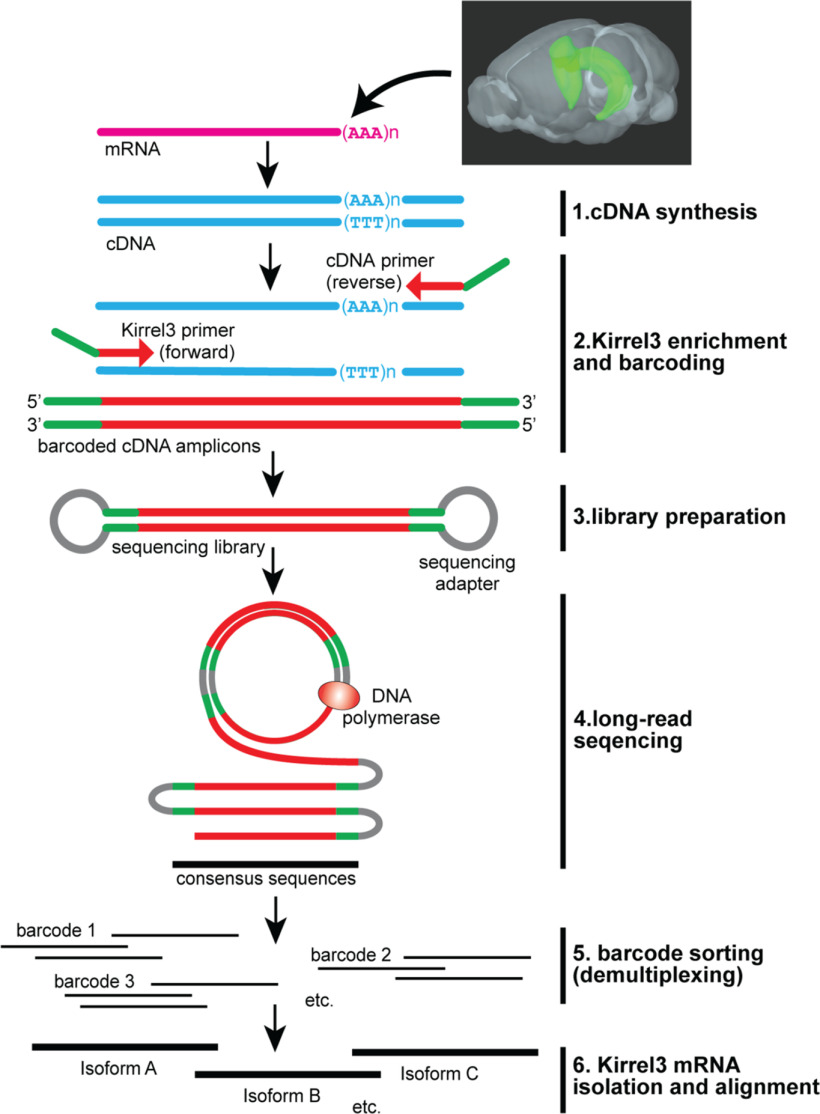
Long-read transcript sequencing. Schematic of the long-read sequencing workflow starting with total mRNA from P14 hippocampal tissue. Pink indicates mRNA, blue cDNA, green DNA-barcodes, red raw Kirrel3 sequences, gray sequencing adapters, and black consensus Kirrel3 sequences. Extended Data Table 1-1 reports the sequence of each bar code used.

Commonly used deep-sequencing technologies rely on short sequence reads (<250 bp) that are powerful assets in defining whole cell or tissue transcriptomes (Corchete et al., 2020). Short sequence reads are suitable to determine relative transcript frequencies and relative exon usage for all genes expressed. However, short-read sequencing is not suitable to directly determine the complete exon composition of transcripts longer than the 250-bp read limit (Leshkowitz et al., 2022). Recent advances in long-sequence read technologies such as Iso-seq (Kuo et al., 2017) or Nanopore sequencing (Y. Wang et al., 2021) can generate millions of high-quality reads of >15 kb that allow full-length transcript characterization. To capture full-length Kirrel3 transcripts, we performed Iso-seq experiments on RNA collected from hippocampal tissue from 14-d-old C57Bl/6J mice (*n* = 4, 2 males, and 2 females). As a synapse specificity molecule, Kirrel3 is not an abundant transcript nor protein. Based on published RNA sequencing data on mouse brain samples (Bioproject PRJDB7898, five replicates), we estimate that Kirrel3 transcripts comprise an extremely small proportion of the overall mRNA content (<0.001%). Therefore, before conducting long-read sequencing, we enriched libraries for Kirrel3 transcripts. We accomplished this via a PCR amplification step using a forward primer matching exon 2 of Kirrel3 and a universal reverse primer with the sequence AAGCAGTGGTATCAACGCAGAGT, which was originally ligated to all transcripts during cDNA synthesis (Fig. 1). In addition, a unique Iso-seq library bar code was added to each sample in this step to allow for simultaneous sequencing of all samples in a single sequencing run. We chose the target-specific primer because exon 2 contains the start codon required for all known and predicted Kirrel3 isoforms. We also conducted Iso-seq on samples from 14-d-old male and female Kirrel3 knock-out mice (Prince et al., 2013) as sequencing and data analyses controls. We find that our modified Iso-seq strategy using a Kirrel3-specific primer generated datasets with a total of 1,394,974 reads including 11,328 Kirrel3 reads (0.8%). Although Kirrel3 remains a small part of the total dataset, we estimate that Kirrel3 transcripts were enriched over 1600-fold on average compared with whole-transcriptome libraries from brain tissue.

10.1523/ENEURO.0253-23.2023.tab1-1 Extended Data Table 1-1 Samples and their barcodes. Each sample represents whole hippocampi of two individuals. Sample cDNA was 5′ and 3′ barcoded using a Kirrel3-specific forward (5′ barcode-CTTCTGTGAAAGGAGCCCTTCT) and a universal reverse primer (3′ barcode-AAGCAGTGGTATCAACGCAGAGT). *KO*: homozygous Kirrel3 knock-out. Download Table 1-1, DOCX file.

### Four independently spliced exons and alternative C termini generate a diversity of Kirrel3 transcripts

**Figure 2.**
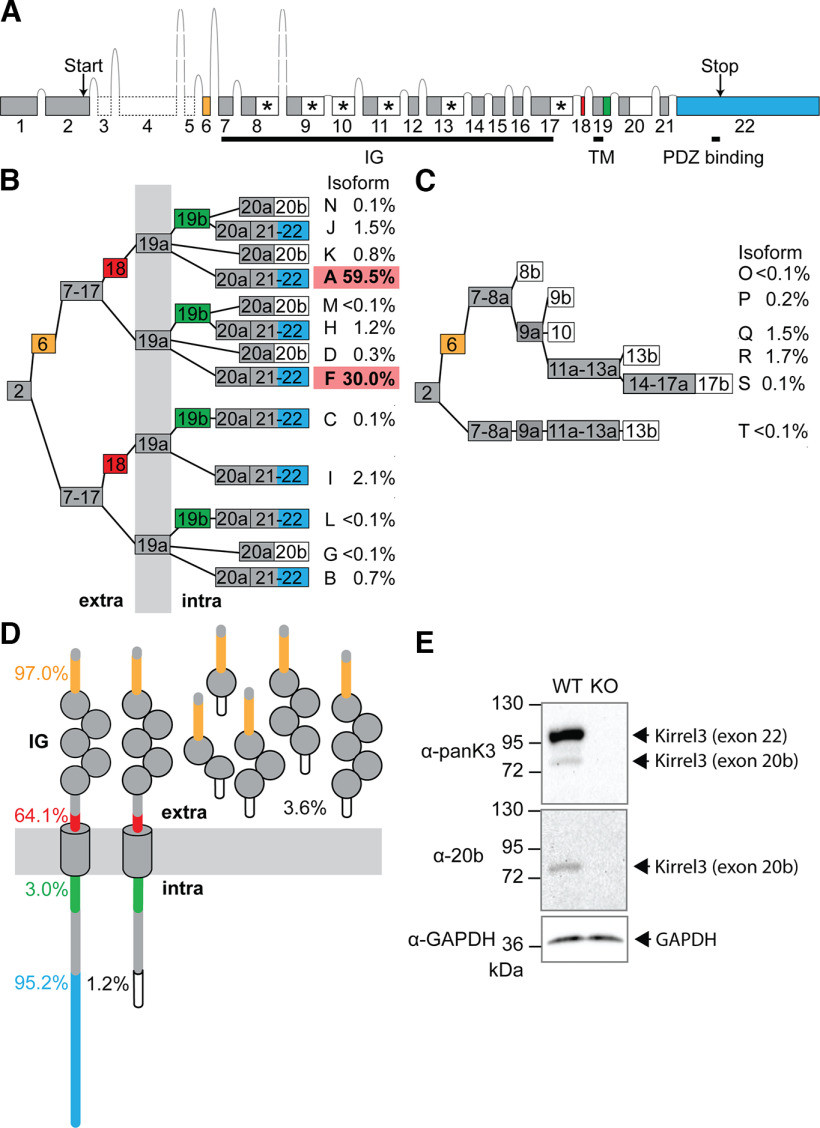
Mouse Kirrel3 gene, transcript isoforms, and proteins. ***A***, Genomic organization of the mouse Kirrel3 gene, with exons (boxes) and introns (lines), including four independently spliced protein-coding exons (yellow, red, green, and blue). White boxes mark exons or exon parts with a stop-codon. Exons producing predicted secreted Kirrel3 isoforms are additionally marked with an asterisk. ***B***, Alternative splicing of Kirrel3 exons is predicted to produce 13 different transmembrane isoforms. Isoforms are given letters, following the example of previously identified Isoforms A–E. Isoform E, the only transcript including exon 5, was not identified in the hippocampus. Exons 8, 9, 11, 13, 17, 19, and 20 are present in transcripts as short (part a) or extended (parts a + b) versions. Percent indicate the relative contribution of a particular Kirrel3 isoform to the total number of complete Kirrel3-transcripts featuring exon 2 and poly A-tail. Exons encode protein segments that are either extracellular (“extra”), intracellular (“intra”), or spanning the membrane (gray vertical bar). ***C***, Six predicted secreted Kirrel3 isoforms (O–T) only comprise extracellular domains. ***D***, Schematic of Kirrel3 protein isoforms. Percent are estimates of how frequent protein domains are present in hippocampal Kirrel3 based on isoform frequencies. ***E***, Western immunoblots from hippocampi from three-week-old wild-type and Kirrel3 knock-out mice using antibodies directed against Kirrel3 amino acids 46–524 (αpanK3), the peptide encoded by exon 20b (α−20b), or house-keeping gene glyceraldehyde 3-phosphate dehydrogenase (αGAPDH). IG: Ig-domain, TM: transmembrane domain. Extended Data Table 2-1 reports the exact sequences for each mouse and human exon as well as SRA files.

In our custom analysis pipeline, 85.8% of transcript reads could be assigned to a sample based on sample-specific barcodes (Fig. 1; Extended Data Table 1-1). The remaining 14.2% of all transcripts either contained incomplete or no bar code and were excluded from subsequent analyses. To uncover new Kirrel3 exons, we screened transcripts of each sample for the presence of any known Kirrel3 sequence, including predicted intron sequences (NCBI Gene ID: 67703). Consistent with our PCR strategy to enrich for Kirrel3 transcripts beginning at exon 2, exon 1 of Kirrel3 was absent in our Iso-seq dataset. Moreover, hippocampal Kirrel3 transcripts neither contained predicted exons 3 and 4 (ENSEMBL genome browser, ENSMUSG00000032036), nor previously published exon 5, but we included them in the overall gene structure shown in Figure 2*A* for completeness (Fig. 2*A*, dashed boxes). Analysis of Kirrel3 transcripts indicates a total of 22 exons, one of which was not previously reported (exon 10 in Fig. 2*A*). These exons are alternatively spliced to generate 19 distinct isoforms in mouse hippocampus. Five were previously identified and designated as Isoforms A–E. Using the same logic, new isoforms are identified with a letter (Fig. 2*B*,*C*). Based on our data, we also estimated the frequency of each isoform (Fig. 2*A*,*B*) and the cumulative frequency of each predicted protein domain (Fig. 2*D*).

10.1523/ENEURO.0253-23.2023.tab2-1 Extended Data Table 2-1 Mouse and Human Kirrel3 exon sequences and databases analyzed. Sequences of the 22 mouse and 21 human Kirrel3 exons. Segments that extend an exon are labeled with the letter “b,” the respective nonextended exons are labeled with “a.” NCBI Sequence Read Archive (SRA) data used to search for mouse and human Kirrel3 transcripts. Download Table 2-1, DOCX file.

Kirrel3 exons found in hippocampal transcripts fall into three groups. The first group contains constitutive exons present in all isoforms found in the hippocampus (Fig. 2*A–C*, gray boxes). The second group are exons that are either fully included or skipped (exons 6, 18, 22). Exon 6 contains a signal-peptide cleavage site and is present in most transcripts. If exon 6 is skipped, an alternative signal peptide is predicted. As a consequence, slightly different extracellular N termini are generated dependent on the presence or absence of exon 6. The third group of exons (8, 9, 11, 13, 17, 19, and 20) are found in transcripts with either extended or nonextended 3′ ends, suggesting that they contain two alternative 3′ splice sites (Xia et al., 2006; Keren et al., 2010; Suñé-Pou et al., 2017). In Figure 2, segments that extend an exon are labeled with the letter “b,” the respective nonextended exons are labeled with “a.” If included in the transcript, six out of the seven b segments (8b, 9b, 11b, 13b, 17b, and 20b) introduce a stop-codon (Fig. 2*A–C*, white boxes) and produce an alternative C terminus in the Kirrel3 protein. Exons 8b, 9b, 11b, 13b, 17b, and the newly identified exon 10 (Fig. 2*A*, asterisks) are predicted to generate secreted proteins with different numbers of Ig-domains (Fig. 2*D*). In contrast, exon 20b is predicted to generate a transmembrane protein with a short intracellular domain lacking the Kirrel3 PDZ-binding domain. Exon 19b does not produce a protein stop, but codes for 25 additional amino acids just intracellular to the transmembrane domain.

The vast majority of isoforms (95%) contain exon 22 and, thus, encode transmembrane proteins with a C-terminal PDZ-binding domain. In contrast, the alternatively generated transmembrane protein with a short intracellular domain lacking a PDZ-binding domain is found in just over 1% of all transcripts. Together with exons 6, 18, and 19b, exon 22 is one of four alternatively spliced exons that appear to be included or excluded from Kirrel3 transcripts independent from each other.

We confirmed our findings by searching published Iso-seq transcript libraries for mouse hippocampus and other brain regions. We found a total of 2237 Kirrel3 transcripts distributed across 21 published mouse Iso-seq libraries (Extended Data Table 2-1) but did not detect additional isoforms. Finally, samples from Kirrel3 knock-out mouse controls produced the expected 5′ untranslated region (UTR) of exon 2 followed by an eGFP coding sequence, which is consistent with how these germline knock-out mice were constructed (Prince et al., 2013).

### Different Kirrel3 protein isoforms are found in brain tissue

Next, we tested whether we could identify distinct Kirrel3-protein isoforms in brain tissue. We initially focused on the existence of isoforms with different intracellular domains because the predicted proteins can be easily resolved by size on a Western blotting. We used a pan-Kirrel3 antibody against the extracellular domain and an isoform-specific antibody that selectively recognizes isoforms containing the short intracellular domain caused by inclusion of exon 20b. We examined immunoblots of mouse hippocampal lysates using the pan-Kirrel3 antibody and detected two bands that run at the expected molecular weight for full length proteins with the long and short intracellular domains (Fig. 2*E*) in wild-type but not Kirrel3 KO tissue. We then blotted the same lysates using the antibody specific to the short intracellular domain only and observed a single band at the lower molecular weight. The results of the immunoblot demonstrate that distinct Kirrel3 isoforms can be identified in brain tissue and that both exon 20b and exon 22 containing mRNA isoforms give rise to proteins in the brain.

### Exons 18, 20b, and 22 do not directly affect Kirrel3-mediated homophilic cell adhesion

**Figure 3.**
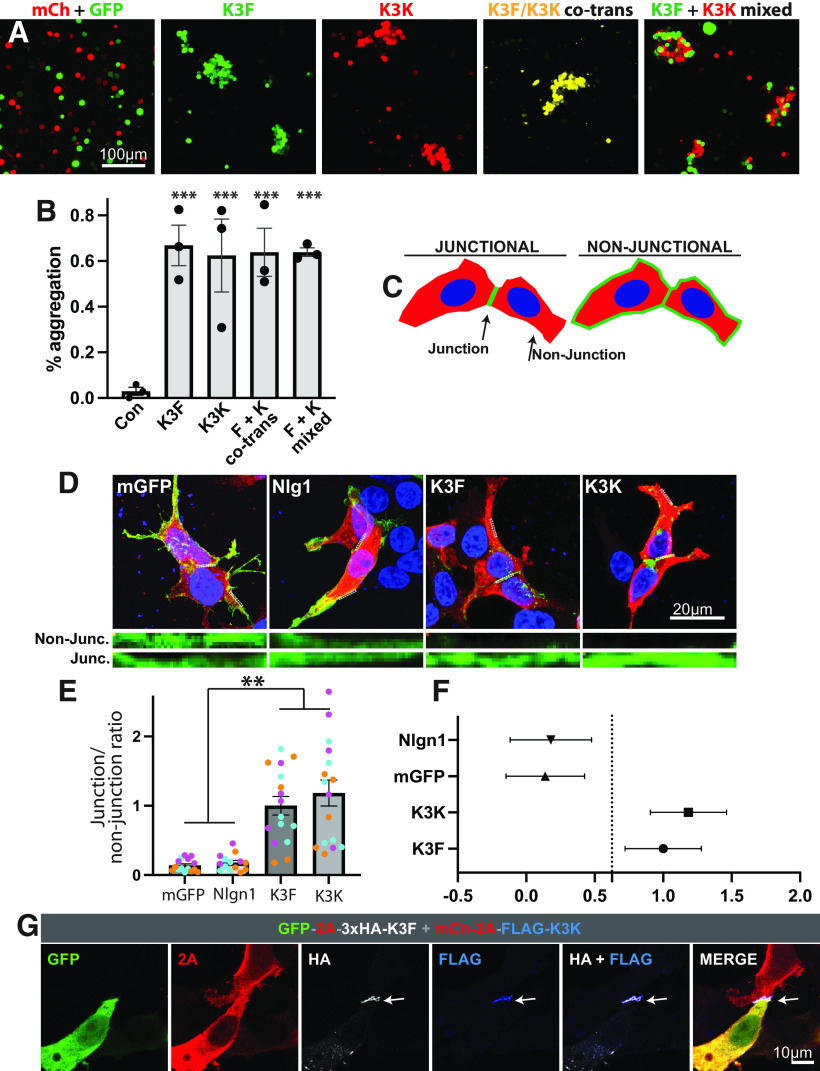
Homophilic binding of Kirrel3 isoforms. ***A***, Example of the CHO-cell aggregation assay. CHO cells are expressing mCherry (mCh) or GFP as a control, GFP-2A-Kirrel3F (K3F), mCherry-2A-Kirrel3K (K3K) or both Kirrel3 isoforms. ***B***, Quantification of CHO cell aggregation assays. *N* = 3 independent trials for each condition. Error bars = SEM *p* = 0.003 for a one-way ANOVA and ***p < 0.001 for each pairwise post-test comparison with the control condition. ***C***, Diagram of the cell junction assay in adherent HEK293 cells. ***D***, Example of the cell junction assay. Note that Kirrel3F and Kirrel3K are both highly enriched in the cell-cell junction, but the control proteins, membrane bound GFP (mGFP) and Neuroligin-1 (Nlg1), are not. ***E***, ***F***, Quantification of the adherent cell junction assay. ***E***, Bar graphs and error bars indicate SEM. Each dot indicates a cell and each color denotes cells from an independent culture. One-way nested ANOVA indicates *p* < 0.001 and **p < 0.01 from pairwise post-tests. ***F***, Same data but graphed as a mean estimation plot with error bars showing SD, indicating that Nlg1 and mGFP are significantly different from Kirrel3F and Kirrel3K. ***G***, Example image showing that K3F and K3K cluster heterophilically in junctions when they are expressed in different cells.

Homophilic, transcellular binding is necessary for Kirrel3-mediated synapse formation (Taylor et al., 2020) and structural studies indicate that Kirrel3-Kirrel3 trans binding is mediated predominantly by its first, most N-terminal Ig-domain (J. Wang et al., 2021). However, accumulating evidence suggests that intracellular domains can allosterically alter the ligand-binding properties of extracellular domains, either directly by inducing conformation changes, indirectly via additional factors, or by altering the stoichiometric assembly of transmembrane receptors (Changeux and Christopoulos, 2017; Ortiz Zacarías et al., 2018; X. Wang et al., 2018; Lara et al., 2019). Moreover, a disease-associated missense variant in the intracellular domain was recently identified that significantly attenuates Kirrel3 trans-cellular binding (Taylor et al., 2020). Thus, we wondered whether the C-terminus choice of Kirrel3 affects homophilic cell-adhesion using an established *in vitro* cell aggregation assay. To test this, we generated expression plasmids encoding cDNAs for isoforms that contain exon 22 (Isoform F) or 20b (Isoform K; Fig. 2*B*). By comparing Isoforms F and K, we sought to also obtain information about whether the selectively spliced extracellular domain encoded by exon 18 affects the homophilic transcellular binding of Kirrel3. Exon 18 is present in Isoform K, but not in Isoform F. We found that suspended cells expressing either Kirrel3 Isoform F or K readily form homophilic and heterophilic aggregates, but control cells expressing mCherry do not (Fig. 3*A*,*B*; Extended Data Table 3-1). We also find that cells co-expressing Isoforms F and K aggregate (Fig. 3*A*,*B*; Extended Data Table 3-1). Similarly, both Kirrel3 isoforms are significantly enriched at cell-cell junctional membranes as compared with other membrane regions when both contacting cells express Kirrel3 (Fig. 3*C–G*; Extended Data Table 3-1). In contrast, neither membrane-GFP nor Neuroligin (a transmembrane protein that does not undergo homophilic binding in trans) are enriched at cell-cell junctional membranes as compared with other membranes (Fig. 3*C–G*). Of note, we confirmed that neither HEK293 nor CHO cells used in any of these assays endogenously express detectable levels of Kirrel3 protein (Extended Data Fig. 3-1). Together, these results show that Kirrel3 Isoforms F and K undergo homophilic trans-cellular binding and suggest that the inclusion or exclusion of exons 18, 20b, and 22 does not directly affect the ability of Kirrel3 to bind itself in trans.

10.1523/ENEURO.0253-23.2023.f3-1 Extended Data Figure 3-1 HEK293 and CHO cells do not express endogenous Kirrel3. Western blotting showing that HEK293 and CHO cell lines used here do not endogenously express detectable levels of Kirrel3 protein. Kirrel3 WT and KO mouse cortex samples were used as positive and negative controls. Download Figure 3-1, TIF file.

10.1523/ENEURO.0253-23.2023.tab3-1 Extended Data Table 3-1 Data analyses. Details of statistical analyses used in Figure 3. Download Table 3-1, DOCX file.

### Kirrel3 isoforms are co-expressed *in situ*

**Figure 4.**
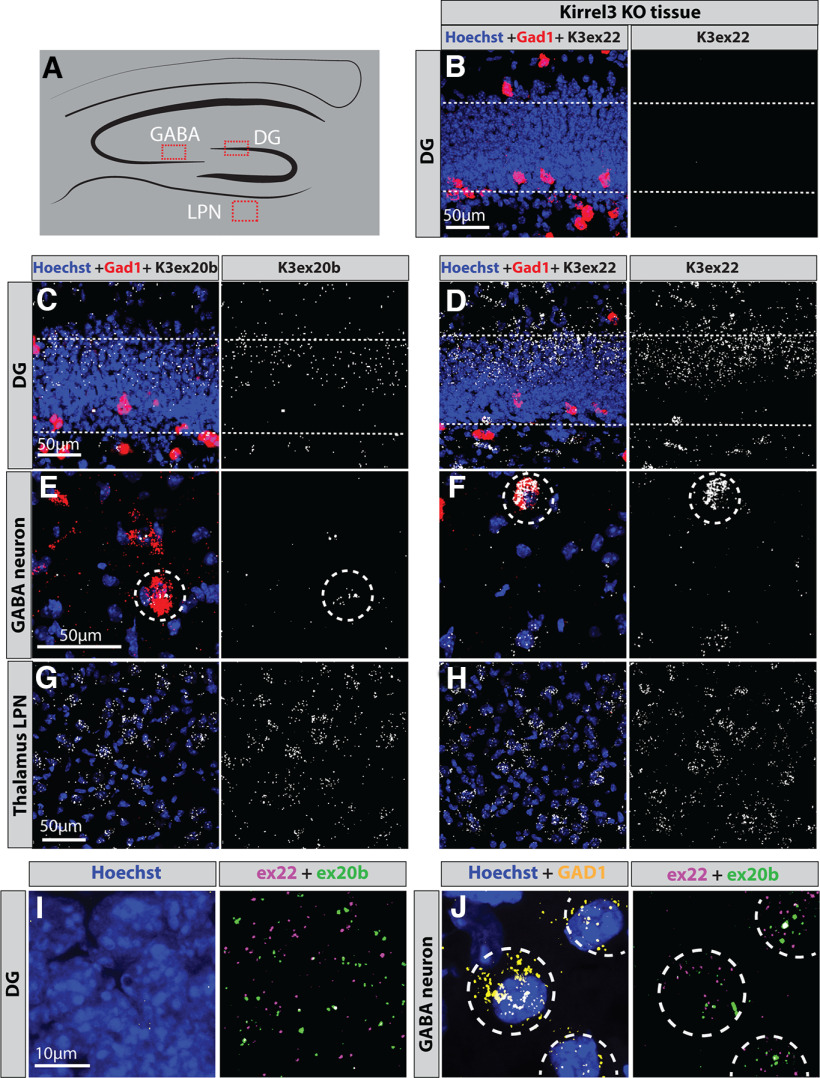
*In situ* detection of exons 20b and 22. ***A***, Schematic of a coronal section through the hippocampus. Red boxes mark areas imaged in B-H. DG; dentate gyrus, LPN; lateral posterior nucleus of thalamus. ***B–H***, Magnified images of regions shown in ***A*** that were hybridized with mRNA *in situ* probes for GAD1 (red) to mark GABAergic neurons, Kirrel3 exon 22 or 20b (white) as indicated to mark specific Kirrel3 isoforms, and the nuclear stain Hoechst (blue). ***B***, Kirrel3 knock-out tissue produces no Kirrel3 mRNA signal using the larger exon 22 probe. ***C***, ***D***, Exon 22 and 20b transcripts are detected in DG neurons of wild-type mice. ***E***, ***F***, Exon 22 and 20b transcripts are detected in GABAergic neurons (white circles) in area CA3 of wild-type mice. ***G***, ***H***, Exon 22 and 20b transcripts are detected in the LPN of wild-type mice. ***I***, ***J***, Simultaneous use of probes for exon 22 and 20b indicate that individual neurons often express both isoforms. Extended Data Table 4-1 reports the HCR probe sets used.

Next, we sought to test whether different cell types or even individual cells express different Kirrel3 isoforms using fluorescent mRNA *in situ* hybridization (FISH). Because Kirrel3 is not an abundant transcript and most of the alternatively spliced exons are very short, we again focused on the two longest alternatively spliced regions, which encode the alternative intracellular domains exon 22 and 20b. We generated hybridization chain reaction (HCR) FISH probes (Choi et al., 2018) selective to exons 22 and 20b and conducted FISH on brain sections of P14 wild-type and Kirrel3 knock-out tissue (Fig. 4; Extended Data Table 4-1). Consistent with previous reports (Lein et al., 2007; Taylor et al., 2020; Hisaoka et al., 2021), we find that Kirrel3 is expressed specifically by DG neurons and GABA neurons in the hippocampus. It is also expressed by many cells in the lateral posterior nucleus (LPN) of the thalamus (Fig. 4). As predicted by our sequencing results, exon 20b is less abundant than exon 22, but we did not observe obvious differences in the expression pattern of both alternatively spliced exons. When we used both probes simultaneously, we found that individual DG and GABA neurons often co-express both isoforms (Fig. 4*I*,*J*). FISH probes to smaller exons did not yield any obvious signal. This is likely a technical issue because Kirrel3 transcripts are not highly expressed and the specific probes are very short. Nonetheless, these findings argue against a cell type-specific use of alternative intracellular domains and suggests that most neurons express a mixture of Kirrel3 isoforms with and without a PDZ-binding domain.

10.1523/ENEURO.0253-23.2023.tab4-1 Extended Data Table 4-1 Fluorescent *in situ* hybridization (FISH) probes. Sequences of probes used in FISH experiments. Download Table 4-1, DOCX file.

### The use of independently spliced Kirrel3 modules is expanded in Hominidae

**Figure 5.**
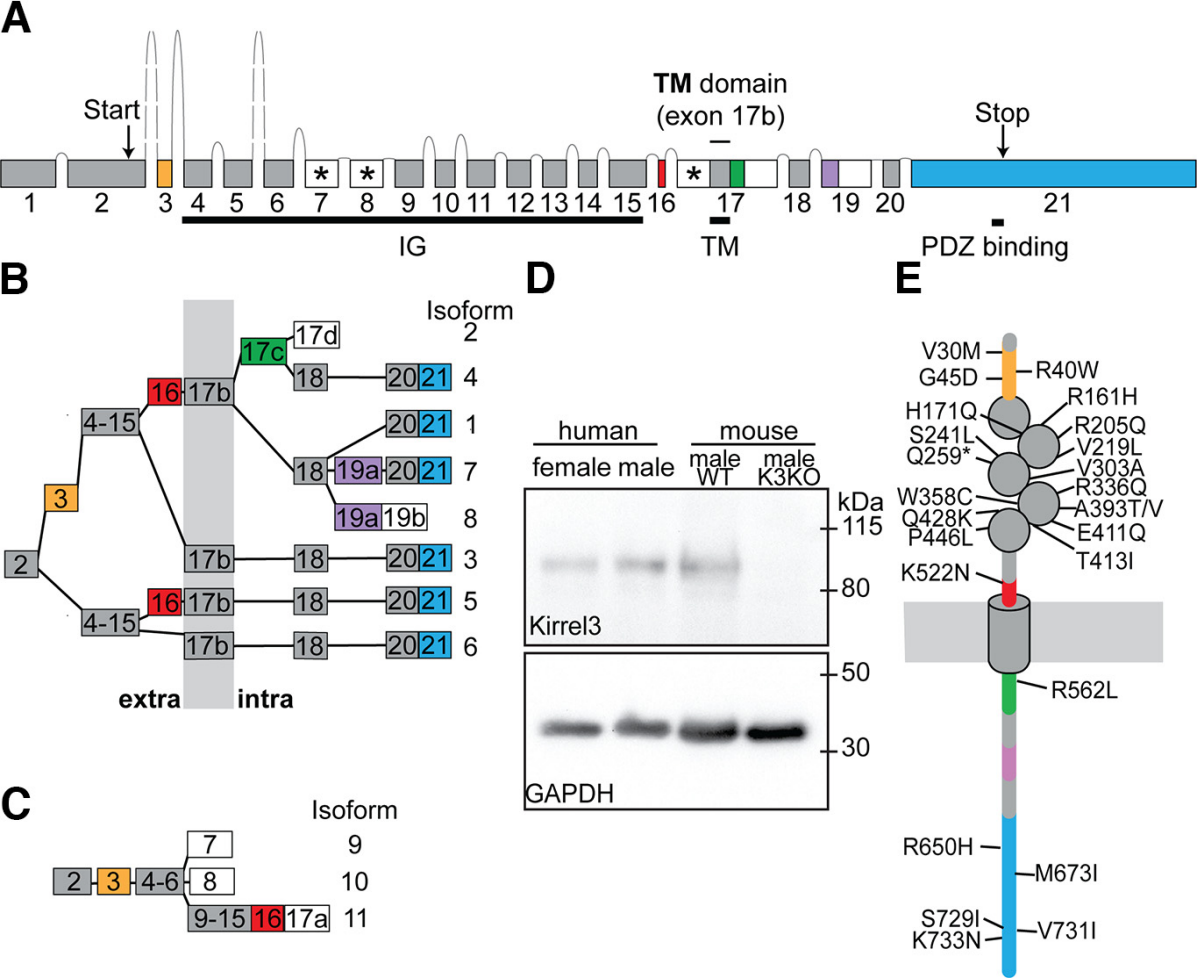
Human Kirrel3 gene, transcript isoforms, and proteins. ***A***, Genomic organization of the human Kirrel3 gene, with exons (boxes) and introns (lines), including five independently spliced protein-coding exons (yellow, red, green, purple, and blue). White boxes mark exons or exon parts with a stop-codon. Exons producing predicted secreted Kirrel3 isoforms are additionally marked with an asterisk. ***B***, Alternative splicing of Kirrel3 exons is predicted to produce eight different transmembrane isoforms. Isoforms are given numbers, following the example of previously identified isoforms 1–3. White boxes indicate exons or exon parts with stop-codon. Exons encode protein segments that are either extracellular (“extra”), intracellular (“intra”), or spanning the membrane (gray vertical bar). ***C***, Three predicted secreted Kirrel3 isoforms (9–11) only comprise extracellular domains. ***D***, Western blot showing that Kirrel3 protein containing human exon 21 is found in brain lysates prepared from male and female postmortem tissue (see methods). Mouse wildtype (WT) and knockout (KO) lysates are used as a positive and negative control. ***E***, Schematic of Kirrel3 proteins with the position of protein domains relative to the membrane (horizontal gray bar) and mutations associated with autism. IG: Ig-domain, TM: transmembrane domain.

To examine the extent to which our findings for mouse Kirrel3 isoforms apply to humans, we searched publicly available human brain Iso-seq libraries for reads containing Kirrel3 exon 2 (ENSEMBL genome browser, ENSG00000149571) and identified a total of 1365 transcripts distributed across 19 published libraries. Similar to our strategy for mouse, we screened the identified transcripts for the presence of any Kirrel3 gene sequence (NCBI Gene ID: 84623), including predicted intron sequences. The screen revealed three human Kirrel3 exons and one exon extension not previously reported (Fig. 5*A*). The human Kirrel3 gene has 21 exons and five alternative C termini, appears slightly more compact than that of the mouse, and does not contain any exons with homology to predicted mouse exons 3–5. Nevertheless, humans have orthologues to the four alternatively spliced mouse Kirrel3 protein coding exons (Fig. 5*A*,*B*). This includes an alternative signal peptide, insertions just before and after the transmembrane domain, and a short intracellular domain that lacks a PDZ-binding domain. Humans also have Kirrel3 isoforms with a completely new insertion (exon 19a) that adds 30 unique amino acids to the intracellular domain. Intriguingly, across all sequenced genomes a homolog to exon 19a was found only in other great apes (including chimpanzees, orangutans, and gorillas), both based on nucleotide and amino acid sequence. Moreover, our search revealed a total of 9 human Kirrel3 transcript isoforms predicted to encode different proteins, including three forms of secreted Kirrel3 (Fig. 5*B*,*C*). The transcript isoforms are designated with a number following the nomenclature of the previously identified isoforms 1–3. Because Kirrel3 transcripts are relatively rare, it is likely that more human isoforms can be found in the future with deeper and targeted sequencing. Nonetheless, we probed lysates prepared from postmortem human prefrontal cortex tissue with an anti-Kirrel3 antibody that recognizes the longest form of Kirrel3 and find evidence that the protein is expressed in human brain (Fig. 5*D*).

Kirrel3 variants may be risk factors for autism spectrum disorder and intellectual disabilities in humans and we wondered whether identified disease-associated variants are located in specific exons or protein coding domains. We searched the SFARI database (Banerjee-Basu and Packer, 2010; Abrahams et al., 2013) and the literature for Kirrel3 missense mutations associated with autism or intellectual disabilities (Bhalla et al., 2008; De Rubeis et al., 2014; Iossifov et al., 2014; T. Wang et al., 2016; Yuen et al., 2016; Li et al., 2017; Kalsner et al., 2018; Guo et al., 2019; Leblond et al., 2019; Hildebrand et al., 2020; Taylor et al., 2020; Zhou et al., 2022). Out of the 25 mutations with the strongest predicted association to disease (Fig. 5*E*), the majority (15) are within the Kirrel3 Ig-domains 2–5. Interestingly, no mutation with a strong disease link was found in the N-terminal Ig-domain despite its prominent role in homophilic transcellular binding. The remaining mutations are found in one of the alternatively spliced protein modules coded by exon 3 (three mutations), exons 16 and 17c (one mutation each), and exon 21 (five mutations), suggesting important roles for these exons in Kirrel3 function.

### Independently spliced Kirrel3 modules appear at branch points in the chordate phylogenetic tree

**Figure 6.**
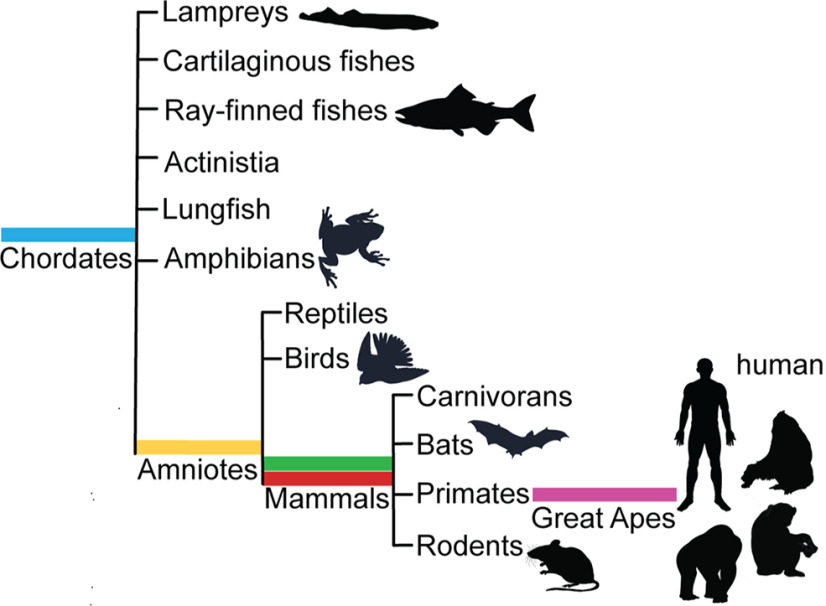
Phylogenetic tree of Kirrel3. The independently spliced protein-coding exons (yellow, red, green, purple, and blue as indicated in figure 5) that produce the Kirrel3 isoform variety first appear at branching points in chordate evolution.

Inspired by the identification of an alternatively spliced Kirrel3 exon only present in humans and their closest living relatives, we examined the presence of all five alternatively spliced protein coding Kirrel3 segments using both nucleotide and amino acid-based searches across all published genomes. PDZ-binding domain coding exon 22 of mice first appears in combination with the Ig-domains characteristic for Kirrel3 in chordates that evolved over 500 million years ago (Holland, 2005; Fig. 6). Mouse exon 6, coding for the Kirrel3 N terminus with a cleavable signal peptide first appears in amniotes, a clade that marks the transition of tetrapods from aquatic to terrestrial habitats ∼300 million years ago (Benton and Donoghue, 2007). Mouse exons 18 and 19b, both encoding protein segments in juxtaposition to the transmembrane domain, first appear together in placental mammals which evolved in the late cretaceous around 90 million years ago (O’Leary et al., 2013). Finally, human exon 19a and its orthologues appear to be the latest addition to Kirrel3 ∼12–16 million years ago when the last common ancestor of all great apes lived (Chen and Li, 2001).

## Discussion

Here, we detect an extensive repertoire of Kirrel3 mRNA isoforms in the mouse brain using a modified long-read sequencing strategy that substantially enriched Kirrel3 transcripts in the dataset. Gene-specific enrichment was an essential prerequisite to discover the extensive alternative splicing of Kirrel3, because expression of this target recognition molecule is inherently sparse and is often limited to a subset of cell types within a tissue. Consequently, we find that bulk and single cell sequencing datasets generally contain limited reads of Kirrel3 transcripts. Moreover, sequencing full-length transcripts was necessary because classic next generation sequencing generates only short reads that identify single or few exons, but not complete exon contigs. The use of long-read sequencing is particularly important for transcripts arising from exon-rich genes like Kirrel3 which features more than twice the exon count of the average gene in both mouse and human (Sakharkar et al., 2004). Because of the relatively high Kirrel3 exon count, it is not surprising that Kirrel3 isoform diversity arises from many different types of splicing events including, exon skipping, alternative 3′ exon splicing (mouse and human), and alternative 5′ exon splicing (human). These three forms of alternative splicing occur at frequencies for Kirrel3 that roughly match the proportions typical for mammalian splice events (38% exon skipping, 18% alternative 3′ splicing, 8% alternative 5′ splicing; Koren et al., 2007).

Importantly, we provide evidence that even rare Kirrel3 isoforms present in our full-length transcript dataset make detectable levels of mRNA and protein in the mouse brain. For example, exon 20b is present in only ∼1% of Kirrel3 transcripts, yet we can detect both the corresponding mRNA and protein in brain tissue using a selective *in situ* probe and antibody, respectively. Moreover, we demonstrate that exon 20b-containing transcripts give rise to proteins. Inclusion of exon 20b produces a Kirrel3 variant characterized by a truncated intracellular domain lacking the PDZ-binding domain typical for Kirrel3 and present in 95% of all isoforms. Despite the truncated tail, Kirrel3 isoforms with exon 20b are still capable of transcellular homophilic binding and can mediate cell-adhesion. The case of Kirrel3 exon 20b illustrates how the study of alternative splicing of synaptic receptors can directly instruct future studies. For example, it would be important to examine how Kirrel3 proteins with truncated tails alter and modulate the function of Kirrel3 isoforms with PDZ-binding domain in forming and maintaining synapses. Addressing this question would also shed new light on how neurons use PDZ-domains, a motif present in various scaffolding proteins known to organize and structure synapses.

In a similar vein, it will be important to study the function of secreted Kirrel3 isoforms that, together, constitute an estimated 4% of isoforms. Secreted Kirrel3 isoforms feature one or more Ig-domains and, thus, are predicted to undergo homophilic binding. It is possible that secreted Kirrel3 binds and sequesters transmembrane Kirrel3 variants and prevents their normal function in trans-cellular binding and signaling. Alternatively, secreted Kirrel3 could activate transmembrane Kirrel3 and trigger intracellular signals that are independent of cell-to-cell contact. Another key finding of the present study is that Kirrel3 alternatively uses several protein-coding exons (exons 6, 18, 19, and 22 in mouse) that fall on both the extracellular and intracellular sides of the protein. Other than the C-terminal PDZ-binding domain present in exon 22, the function of the other protein-coding exons remains unknown. However, it is reasonable to assume they are essential for Kirrel3 activity because mutations in the equivalent human domains are associated with disorders. Moreover, these domains first appear at critical branching points in chordate evolution. In this context, the newly discovered intracellular Kirrel3 exon encoding 30 amino acids present only in humans and other great apes is of particular interest as it could serve a synapse-related function unique to Hominidae.

## Synthesis

Reviewing Editor: Benjamin Deneen, Baylor College of Medicine Decisions are customarily a result of the Reviewing Editor and the peer reviewers coming together and discussing their recommendations until a consensus is reached. When revisions are invited, a fact-based synthesis statement explaining their decision and outlining what is needed to prepare a revision will be listed below. The following reviewer(s) agreed to reveal their identity: Andrew Garrett.

The reviewers found the paper to be of significant interest, requiring only minor revisions.

Please see the comments below and we welcome your revised manuscript.

## Author Response

Synthesis of Reviews:

The reviewers found the paper to be of significant interest, requiring only minor revisions.

Please see the comments below and we welcome your revised manuscript.

We thank the reviewers for their time and thoughtful comments to improve our manuscript. We addressed each point as described below.

Reviewer 1

In this manuscript, the authors performed targeted long-read sequencing to find Kirrel3 splice variants expressed in the mouse hippocampus, identifying 15 new isoforms. They verified protein expression from rare transcripts (i.e., those including exon 20b), found that alternate exons are expressed in the same cell types by in situ hybridization (i.e., no obvious cell type specificity to exon choice) and showed that variants with changes in the extracellular and cytoplasmic domains are still capable of homophilic interaction in a cell aggregation assay. Finally, they mined existing sequencing data to identify previously undefined transcripts expressed in humans, and described when alternatively spliced exons arose in evolutionary history.

Kirrel3 is an interesting cell adhesion molecule with important functions in neural development and is a strong candidate for involvement in autism spectrum disorder. Overall, I found the manuscript to be well-written, the experiments well-controlled, and the results appropriately interpreted. I was left with some questions that could be answered by additional experiments (described below), but these are not required to address the core claims of the paper.

1.) The cell aggregation assay demonstrated that both isoforms F and K are competent to engage in homophilic interactions, but do they interact in trans with each other? Testing aggregation between the two would address this.

Because the extracellular domains are so similar, we suspected that Kirrel3 isoforms F and K would interact in trans with one another, but this had not been formally tested. Based on reviewer feedback, we expressed isoforms F and K in different cells and conducted the trans-binding aggregation assay. We now show that cells individually expressing isoforms F and K aggregate to one another, suggesting that isoforms F and K bind in trans. Moreover, because our new data suggest that individual neurons express multiple isoforms of Kirrel3 (see reviewer 2 comment 4), we also tested if cells co-expressing isoforms F and K aggregate and we find that they do. This new data is presented in revised figure 3A,B.

2.) The predicted secreted isoforms are intriguing. As mentioned in the Discussion, these could act at a distance to “activate” Kirrel3 or to mask adhesion. This latter possibility could also be tested in the cell aggregation assay by co-expressing, e.g., isoforms A and R.

We have a limited amount of purified protein of the Kirrel3 extracellular domain and we tried adding this to the cell aggregation assay. Qualitatively, it appeared to diminish cell aggregation but working out stoichiometric conditions and purifying more secreted Kirrel3 protein will take considerable time. This is a very interesting direction that we are pursuing in the future but we hope the reviewers agree that it is not required for this initial publication.

Points that should be addressed in the text:

1.) Transcripts including exons 3-5 were not detected, nor isoform E. Does isoform E include these exons? Are exons 3-5 absent in humans?

Mouse isoform E, which is reported in NCBI but is not present in our mouse hippocampal data, includes exon 5 but not exons 3 and 4. Exons 3 and 4 are predicted in the ENSEMBL genome browser but not found in any confirmed transcripts. We did not find any human transcripts that included orthologous exons to mouse exons 3-5. We revised the text and modified Figure 2 to state these points more clearly.

2.) Page 15, line 10 - “This finding suggests an important role of this segment and Kirrel3 in the evolution of the brain.” This overstates the correlation and should be toned down or removed.

Thank you. We removed that sentence.

Reviewer 2

This study reports on the repertoire of Kirrel3 mRNA splice variants that are expressed in the mouse hippocampus, and that diversify to some degree across phylogeny. The results are fascinating and significant. And the experiments are well done and clearly presented. However, there are a few points raised by the authors that require clarification:

1. Figure 2 shows the alternative transcripts and isoform organization, but the diagrams are confusing. Fig 2A would benefit from a more conventional representation of alternate exons, rather than simply color coding a single box. The alternate exons that produce the secreted forms should also be demarcated. In Fig 2b, only a subset of exons are shown on the left but they should all be included. Are exons 3-5 excluded from spliced? (the diagram for the human Kirrel3 includes exons 3-5. The IgG-encoding set could be explicitly labeled as IG: 7-17.

The alternative transcripts and isoforms of Kirrel3 are extensive and complex but we made several changes to Figure 2 to improve the clarity the figure based on the reviewer comment. We are showing all isoforms identified in our data. Because exons 3-5 were previously predicted to exist but not found in our data or any other data sets we searched, we changed those exons from a solid outline to a dashed outline. We directly labeled exons that make up the immunoglobulin-like domains with the abbreviation “IG” as well as the transmembrane domain (TM) and PDZ-binding domain (PDZ binding). We indicated exons that sometimes generate a pre-mature stop codon to produce predicted secreted isoforms with asterisks. We maintained the grey and white color coding to indicate alternative 3’ or 5’ exon extensions as this is standard practice in the field (Xia, Bi et al. 2006, Koren, Lev-Maor et al. 2007, Keren, Lev-Maor et al. 2010, Sune-Pou, Prieto-Sanchez et al. 2017) and brightly colored exons (orange, red, green, blue) indicate exons that appear at key points in evolution. We revised the figure legend for Figure 2 to clearly describe the annotations and updated figure 5, describing human transcripts, to match.

2. The composition and significance of ’extended exons’, ie. exon 19b vs 19a should be described in more detail, by introducing the concept in the results, and elaborating in the discussion. I’m unfamiliar with these forms - do extended exons commonly contribute to alternative transcripts/splicing. How do they differ from alternate exons? And they contribute to a very small portion of the Kirrel3 isoforms - is this typical of extended exons?

Alternative 5’ splicing and 3’ splicing of exons are the second and third most common forms of splice events in mammals after exon skipping (38%), with 8% and 18%, respectively. The revised manuscript includes these important facts and citations that were missing in the original version.

3. In Figure 3, the authors test ‘if the C-terminus choice of Kirrel3 affects homophilic cell-adhesion using an established in vitro cell aggregation assay’, but only test for Kirrel3 isoforms F with F, and isoform K with K. But a test for isoform-specific homophilic adhesion is lacking. A new experiment testing Kirrel3 isoform F binding to isoform K would further discriminate the influence of the C-terminus choice, and assess the contribution of exon 18 to isoform-specific homophilic adhesion (present in K, lacking in F).

See reviewer 1 comment 1 above

4. In Figure 4, the authors ask whether ‘individual cells express different Kirrel3 isoforms using fluorescent mRNA in situ hybridization (FISH)’ but do not directly test whether 20b- and 22-containing transcripts are co-expressed within a given single cell. Additional multiplexed smFISH experiments would strengthen this section (triple staining, including the gad1 probes), and support the authors’ assertion that the mRNA isoforms are co-expressed.

The revised manuscript contains this additional in situ information. We changed the fluorophore on one of the isoform specific probes so that we could use the probes for isoforms 20b and 22 on the same sections. High magnification images in revised figure 4I and J strongly suggest that individual GABA neurons and DG neurons co-express both isoforms. The figure and the results section have been updated to reflect this additional information.

5. The study claims that the human Kirrel3 transcripts are ‘protein-coding’. Can the pan-Kirrel3 antibody be used in western blots in human tissue or cells for human Kirrel3 to support this claim?

To test if we can detect Kirrel3 protein in human brain tissue, we obtained samples (one male, one female) of human prefrontal cortex that we could prepare for a Western blot. We first tried to detect human Kirrel3 protein with the monoclonal pan-Kirrel3 antibody used previously that is widely available from NeuroMab. Unfortunately, that antibody did not prove sensitive enough for detecting human protein. However, we then tested a non-commercial, polyclonal antibody reported in a 2015 eLife paper. This antibody can only recognize a peptide present in exon 22 (the longer intracellular domain) but it clearly identified a band in both human samples and wildtype mouse brain that was absent in Kirrel3 knockout mouse brain. This antibody is a finite resource but it appears to be very specific (there were no “background” bands in any samples) and indicates that Kirrel3 protein is expressed in human brain tissue. This new data is presented in the revised figure 5 and is described in the revised results and methods sections.

Minor comments:

a. The first portion of this sentence is missing: ...” Leblond, Cliquet et al. 2019, Hildebrand, Jackson et al. 2020, Taylor, Martin et al. 2020, Zhou, Feliciano et al. 2022), understanding alternative splicing of Kirrel3 may facilitate the study of disease mechanisms that involve this synaptic gene.

This sentence technically was complete but it had a huge list of citations in the middle that broke up the flow. We rewrote the sentence so that the long reference list is at the end of the sentence for improved readability.

b. My understanding is that the HCR 3.0 system uses pairs of split probes. Are the probe sequences in the Extended Data, Table 4-1 paired? If so, the pairs should be defined, ie. probe 1a, 1b; 2a, 2b.

We amended the Extended Data Table 4-1 to include this information.

c. This sentence is not clear: “and a universal reverse primer to a sequence added during the prior cDNA-synthesis step (Figure 1).”

We edited that sentence in the revised manuscript to improve clarity. The new sentence reads; “and a universal reverse primer with the sequence AAGCAGTGGTATCAACGCAGAGT, which was originally ligated to all transcripts during cDNA-synthesis (Figure 1).”

d. The images in Figure 3C-F show mGFP protein and Neuroligin protein localized to both junctional and non-junctional regions. Therefore, this statement should be clarified to emphasize that Kirrels are significantly enriched at cell-cell junction compared to other membrane regions: “In contrast, neither membrane-GFP nor Neuroligin (a transmembrane protein that does not undergo homophilic binding in trans) are enriched at cell-cell junctions (Figures 3C-F).”

These sentences were revised for clarity.

e. Does the cell line used for suspended cultures endogenously express Kirrel3?

We tested if either cell line used in the manuscript (HEK293 or CHO cells) endogenously express Kirrel3 by Western blot using the highly sensitive custom antibody used for human brain lysates. We find that neither cell line expresses detectable levels of Kirrel3 protein and this new data is presented in extended data figure 1. Moreover, our data is consistent with RNA sequencing that has been reported by others for these lines. Analysis of HEK293 (GEO: GSE242551) and CHO cell (GEO: GSE157729) transcriptomes revealed that both heterologous expression systems are virtually free of endogenous Kirrel3 transcripts (HEK293: 43 out of 217,750,383 transcripts; CHO: 5 out of 130,368,530 transcripts) (Malm, Kuo et al. 2022).

Additional references:

Keren, H., G. Lev-Maor and G. Ast (2010). “Alternative splicing and evolution: diversification, exon definition and function.” Nat Rev Genet 11(5): 345-355.

Koren, E., G. Lev-Maor and G. Ast (2007). “The emergence of alternative 3’ and 5’ splice site exons from constitutive exons.” PLoS Comput Biol 3(5): e95.

Malm, M., C. C. Kuo, M. M. Barzadd, A. Mebrahtu, N. Wistbacka, R. Razavi, A. L. Volk, M. Lundqvist, D. Kotol, H. Tegel, S. Hober, F. Edfors, T. Graslund, V. Chotteau, R. Field, P. G. Varley, R. G. Roth, N. E. Lewis, D. Hatton and J. Rockberg (2022). “Harnessing secretory pathway differences between HEK293 and CHO to rescue production of difficult to express proteins.” Metab Eng 72: 171-187.

Sune-Pou, M., S. Prieto-Sanchez, S. Boyero-Corral, C. Moreno-Castro, Y. El Yousfi, J. M. Sune-Negre, C. Hernandez-Munain and C. Sune (2017). “Targeting Splicing in the Treatment of Human Disease.” Genes (Basel) 8(3).

Xia, H., J. Bi and Y. Li (2006). “Identification of alternative 5’/3’ splice sites based on the mechanism of splice site competition.” Nucleic Acids Res 34(21): 6305-6313.
