# Supplementary material for: Modular Splicing Is Linked to Evolution in the Synapse-Specificity Molecule Kirrel3
Source: eNeuro. 2023 Dec 4;10(12):ENEURO.0253-23.2023. doi: 10.1523/ENEURO.0253-23.2023 (PMC10698715; doi:10.1523/ENEURO.0253-23.2023)
Supplement: Extended Data Table 1-1 — Samples and their barcodes. Each sample represents whole hippocampi of two individuals. Sample cDNA was 5′ and 3′ barcoded using a Kirrel3-specific forward (5′ barcode-CTTCTGTGAAAGGAGCCCTTCT) and a universal reverse primer (3′ barcode-AAGCAGTGGTATCAACGCAGAGT). KO: homozygous Kirrel3 knock-out. Download Table 1-1, DOCX file. [file enu-eN-NWR-0253-23-s03.docx]

**Extended Data, Table 1-1**

| **Sample** | **Barcode** |
| --- | --- |
|  |  |
| wild type females #1 | ACAGTCGAGCGCTGCG |
| wild type females #2 | ACACTAGATCGCGTGT |
| wild type males #1 | CATATATATCAGCTGT |
| wild type males #2 | TCACGTGCTCACTGTG |
| *KO* females | CACTCGACTCTCGCGT |
| *KO* males | CACGCACACACGCGCG |

**Extended Data, Table 1-1: Samples and their barcodes.**  Each sample represents whole hippocampi of two individuals. Sample cDNA was 5’ and 3’ barcoded using a Kirrel3 specific forward (5’ barcode-CTTCTGTGAAAGGAGCCCTTCT) and a universal reverse primer (3’ barcode-AAGCAGTGGTATCAACGCAGAGT). *KO*: homozygous Kirrel3 knock-out.
