# Supplementary material for: Modular Splicing Is Linked to Evolution in the Synapse-Specificity Molecule Kirrel3
Source: eNeuro. 2023 Dec 4;10(12):ENEURO.0253-23.2023. doi: 10.1523/ENEURO.0253-23.2023 (PMC10698715; doi:10.1523/ENEURO.0253-23.2023)
Supplement: Extended Data Table 2-1 — Mouse and Human Kirrel3 exon sequences and databases analyzed. Sequences of the 22 mouse and 21 human Kirrel3 exons. Segments that extend an exon are labeled with the letter “b,” the respective nonextended exons are labeled with “a.” NCBI Sequence Read Archive (SRA) data used to search for mouse and human Kirrel3 transcripts. Download Table 2-1, DOCX file. [file enu-eN-NWR-0253-23-s04.docx]

**Extended Data, Table 2-1**

**Mouse Kirrel3 gene (NCBI Gene 67703)**

| exon |  |
| --- | --- |
| 1 | CTGCTTCCCAAGTTGCTGGCTTTCTGCAATCTCTTGATAGCTACCATTCCCGGTGGCTTTGTGCTCTGAGACTCCGTCCTCTGCGTATCACCCCCTAGATGGTGAGGCTGCCTGCAGGGATGGTGCTCACCCTCCTTGCCCCTCTTGGATGCGCACTACCGGTCTAAGGACTCTCCAAGCTTCTCCATCAGGTGGAGCCGTCCTCTCATCGCCTGCCTCCCTCCTCCCTCGCAGCGAACTGCGAGCAGGCCATCCTACAGAGCCGGGAGGTTAACCGAGCCCCCGGAGGAGGTGAGGCCGCCAGCCTGCGCGGGCGGAGATGGCGGGGCCTCCGTCGGGGCCGGCGCCGGCGCCAGAGCCCGGCAGCCTGTGGGTCCTGCAG |
| 2 | CTTCTGTGAAAGGAGCCCTTCTGTCACTCGTCACTCGCTTGCTCGCTCGCCCGCTCGCTAGCTCGTTCGCTCGCTGTGGGAGGAGCCCGCCAGAGGAAGCCGTGTGCCTGGGATGCCAAGAACCAGAGAATGGATCTGCTCCGAGTGGGCACATTGCTAAGGATCCCGGCTTCCCGAGGCGACTGAAAACAAGCATTTGGTTTCGGCTGCCTGCAGATACCCGGAGACACAACGAGACCTAAGCGGACCAGAGGAGGGACAGACCGACTGACAGATAGATGGTCGGCGCGAACCCTGGAGGACCGGCGGCGGAGGCTGAGCACCGCGAGCCCAGCCGCCGCGCTTGAAGAGAAACTAACTGCACACCCAAGTTGCCCGCCGGCTGCCCGCGCGCTGAGGAATGAGACCTTTCCAGCTGGATTTGCTCTTCCTCTGCTTCTTCCTCTTCAGTCAAG |
| 3 | GTAAAGAATAAGAATTCTGGCGGTATACTTCCAGAAGTCGGTAAATGTCAATATTGGATAAGTGTTTCCTTTATATCATGGCAATTTAGCATAAGCTGCTTGGAAACAAATATCTTCCGCAAGCATACAGTGTTTCA |
| 4 | ACATACCCAGAGCTATGTTTCCATGATGGTTCTACATCCTGTCAAGTGCTCAATCAAGATTAACCATTCTGCCCCTGCAGCACCTGATTCCTTGAAAATCTGCCACCCTCACTAAGCACCAGCCAGCCTCTCAGACACTTCCCTATTCTGGTGAGAATATCTTCAACTCCACTTTGTAGTTGAGGAACTGGAATCTCATTGGGGGTAATTTATTTACCCCACATATAAATAATAGGGGTGAGCCTTCTGTCTCCAGTGGAAATCTCCCCAGCCTTCTGAGCTCTTCTGAGTCAGCCCAGCAGCAGACAGACTCTTACCTTCCCCAGACTCTGAGTCAGGCTTCAGTGCTCCCAGAGAATGAATTTCCTGCAAATCAATCCTGGATGCAGTTATTATTTTATAGGGATGTTGTAAGTTGATAAGGAAGCTGTTATATGCTAATAGCTATTGTAATGTTGTTCTTAAGGCCTATTACTTCTGCTGGTTTGTATTTTAATGTGTGCAAAGGTGATTACAACTTTAATATGAATGTTAAACAGATTAGCTGATTTATATGTAAAATAAATAAATAAATGGAAACTCATACTCTCA |
| 5 | GACTTCTAAGCCTGTGAAGGATCTTTCCAACTCCCTTCAACTTTTCCAGCTCATTTGTCTTATGCCTCCATGCTGACCTGATGGCTCACCACATGTATTTG |
| 6 | AGCTTGGCCTCCAGAAGAGAGGATGCTGTCTGGTACTGGGCTACATGGCCAAGGACAAGTTTCGGAGAATGAATGAAG |
| 7 | GTCAAGTCTACTCCTTCAGCCAGCAACCCCAGGACCAAGTGGTGGTGTCAGGACAGCCAGTGACTCTGCTGTGTGCCATCCCTGAATATGATGGCTTCGTCCTGTGGATCAAAGATGGCTTGGCTCTGGGTGTAGGCAGAGACCTCTCAA |
| 8a | GTTACCCCCAGTACCTGGTGGTGGGGAACCACCTCTCAGGAGAGCATCACCTGAAGATCCTGAGGGCTGAGCTTCAGGATGATGCCGTGTATGAGTGCCAGGCCATCCAGGCTGCCATCCGGTCCCGCCCTGCACGCCTCACCGTCCTGG |
| 8b | GTAAGTCACCTACATACAGGTATGCAGGGATGGCATAAGGACTCTGAGAACCTCCAACAGTTCAGCTACTGGAGAGTCCTTCTACAGAGCTTGTCATGGGAGGCTCACCTTTGGATTTCATTGCATCTGGCTCCATCTACTTCTGCCCTCTCCTCTTCCCATCCAAAGGGGACAGGTAAAAGTCCCCCACCTTAGGAAATCATATTGCCACCAACCTATTATTGTCCTACCTAACCCATGTATTTGGCTGTACGTCGGGAGTAGATGAGGGTCCTAGGTATTCTACAGTGTAAATGAGGCCAGGCCTGCTGCAGCAGTGCCTGTCTGGAAAAGAATGTGACAGAGACTGAAAGACCCTTCTTCCAGAGATGACCTCCAAAAGGATCAGAAAAGCTCTATATGTCCTGACCCCCTCCTAAAAATGAGTCTCAAGTTTGGTCCTCCATGACTTCAGTGTGCAAAGACCCTTGTTCCAGAGGCCTTCAACCCTGGTCCCCTTGGAGTCCTGGTTCTCAGGACATCCCAGTAAGATTTCATTTCATGTCCTTATTCTTACAATTTTCATTCTTGCTTTTTGTTTCCCATTTTTTATTCAAATGTCACTGGGACAACCTTCCTGAAGAGGTCCCCACTCCTCCATTAAGGGCAGACCTGCCAGAAGATATAGAGAGGTGGGGACTTGTTCAATGACACCTTAAACTTCCCAATTTTAGTTAAGAGTTCGGCCTTGTCACCTCCTTCCTCTGGCCACGTTTTCCAGGGAAGGGGGCTGTTGGAAAACAGTTTGTTCACATACATGATAATCATTCATAATTGC |
| 9a | TGCCACCAGATGACCCCATCATCCTAGGGGGGCCTGTGATCAGCCTTCGGGCAGGGGACCCCCTCAACCTCACCTGCCACGCAGACAATGCCAAGCCTGCGGCTTCCATCATCTGGCTACGTAAAGGAGAGGTCATCAATGGAGCCACCTACTCCAAG |
| 9b | GTGAGCCTGAGGGAGAGAATGGGGGTGCAGGATGGAGAGTGAAACTAAAGATAAGGGGAGACAAGGGTGGTGGTTTCAGGTGCATAGGGACAAGCTCTGCCTCTCTTCTTGTGGAAGTGCTTCTGTTGTTGCTTCACTCCCCCTGTGTGTGCTTGTTTGCTTGCTTGTTGCTTGCTTGCTTGTTGCTTGCTTGCTTGTTTGTTTCATTGCTCACTTTAGTCCTCAGGCCGTTTCTTCCACTACCCTCCCCTTCCCAACCGCCCCATCCTATTCCCCAACTAGAATAACTAGAGGTGAAAACCCTAGAACAAGGTATTTCAGAAGAGGAGACGAGCCAGAAGCAGCCAGGCTAGGATTGCCACAACTAATTCGTGATCCTATGAATAACTGCCTAAATGCCCTGTCTGCCACAAAACCTCCTTAATTGAATTAAGTCTCAAGAGAGTAAAGATAGAGCCTTTGGAAGGTGGTTATGTCATGAGGGCGTCTACATGAATGAAATTAGTGTCCTTTTAAAAGAGGCCTAAGCACCAAACCCAGACACCATTGCATATGCCAGCAAGATTTTGCTGAAAGGACCCTGATATAGCTGTCTCCTGTGATGCTATGCCAGTGCCTGGCAAATACAGAAGTGGATGCTCACAGTCATCTATAGGATGGAACACAGGGCCCCCAATGGAGGAGCTAGAGAAAGCATCCAAGGAACTGAAGGGGTCTGCAACCCTATAGGTGGAACAACAATATGAACTACCCAGTACCCCCAGAGCTCGTGTCTCTAGCTGCATATGTAGCAGAAGATGGCCTAGTCGGTCATCATTGGAAAAAGAGGCCCCTTGGTCTTGCAAACTTTATATGCCCTAGTACAGGGGAACACCAGGCCCAAGAAGTAGGAGTGGGTGGGTAGGGGAGCAGGACAGGGGGTGGGTATAGGGAACTTTTGGGATAGCATTTGAAATGTAAATAAAGAAAATATCTAATAAAAAATCTATTTGGAAAAAAATTTTAAATAAAATAAAAGAGGCCTAAGAAAGTCCCCTGTCCTTTTCACCATGTGAAGATGGAAGAGGACAGCCACTAACAAAAAGGCATCTGACCCTTGCCATACACTGGGTCAGTCTGCCCTGGTCTTGGGCTTCTTACCCTCAGGGACTATGAAAAAAAAAAAAA |
| 10 | CTCATCTGGAATCCACAACCTATGCTTCCCTTTTTTCTCTGCCTTGTAGTCCAGAGCAGGATCCTCAGGACCAGCAACCAAAAATATCAGATCTCTCTACAGCCCAGAGCCCACCAAAGCTCATCAAGCTATCCATCCCTAAACCTGTGCAGCCCCTACCCTGCCTCATCCACCCCTTTCCCCACAAACCACAGCAAAGGTTCATCGCACACTCTCTGCTCCACTCCCAGTGGCCTGGTGTGGCATGGCTTACCATGTCCCCTCCTCTTGTGAACTGTGAATTACTACTATGGTCGTGGCTTCTACATGTGTTCATGAATACAGCCAGAAATAACCAGTGCCACATACCTCCCCAGACGACTTTTATGTCATTCCAGGTGTAGTTTACGTTCTGCTGAGACTTCCAGATGAAGGAAACCTTGGAAGTCCATTGGCCCTTTTTATCTAGGGACAGAAAACTGGGATCTTGAAGGGTGGTGCCTGGACTCAGTTGACTTCTAGCAAACTTAGAAAACCTAGGTTTTCATCTTTTGGGGGGGGGGCTCTCCCAGACAGGCTCAGATCCCTAAGGTCCCCCAAACACCACCAGATGCTATATCAGGATGCCAGGAAAAGCAGCCTCCTGGCTCTGCAGTTGGGCCATAACCTTCCAGGACCCTGACTTGGGGCCCTCAGTTTTAGAGGGTGACAAGGTACATGCTTGTTCTTTAGTTCTGATCCCCAGCTGGCTCCTCCCCATAGGTCCTTAACAGAAGAAATGTCTTTTCCATCAGCAGAGGATGTGAATAAAAGGCCATTCCTATTCTAAGGATGGTTCTGTCCCATCTCAAGTTGGCTCTGCTGCCAAAGGCCACAGATCAAAGCTTGGCGTTACCACCCAGATCCTGGGGCCAGATTCCCAAACAAATGGCTTCTCTTGGGCCTGAATCAACCCCAGGAACAATGCTTTCTCTTGGGAGATAAGTCTGCTGCTCACATCCTGGGTGGAGCCTCACTGTGAGGATGTCAAGAAAGGCCAAGGTCATCCAAGAGAGACTGGGAAGGAGTAGGGTGTTTCTACATTGGAGGTGCCTGTCCCGGGTCTTTCTGAAGATAGTCCAGCCTGACCCTCCCAGGATACAGAAGAGAAGAGGATACACCTCATCTAGTGAAGACGGGGTCAGGATCACCCTATGAGACCTATTCCAGGGTAGCATACTCAAAAGGCCACTCTCTCCCACACAAGGAAGACAGCTCAGCCAGCCTGGCACATTGCCTTTCTGGAAAGGAGACTGTGTGCCTTTCCTTGCCCAGTTCCACCCTTATTTGGACTGTCACAAGGAGAATGGCATCTGGGCCAGCTGCCAGACCCTGAAGGGCCATGCCAGGCCCGGTTCTCTGTAATACTAGGCAACTGATTTCCTGGGCTCAGTTTCCTCCTCTGAACATGAAAGAGCTGGAAGAAACACTCAGGAAGTGCTCCAGCTGTGACAGGCTGCAAACCCATGGACTGCACCTATCTTAATAGGAGGAGCAGCCTGGCCCAGGAGAACGATAGGACCCAAGCTGAGGGGCTGTCTGAGACTGACCCCCACTCCCCATCTCCTTCCAGGAGTTCAGTTCAGCCAGGCAGTTTCTTCTCCAGAACTGCAAAGACAAGGGTCATAAGCTAAATAGGACTTCTGACTTACTTACAAAGACAGAGTAGCTGGGACTACACAGTACACACACACACAACCACTCAGACAGGGTCCACAGGAAAGTCTCCAGAACTGGCAGCTTGGGATCTAAGGTCTTCAGGATTTGTCACATTGTGGTAGTTAAGCACACTGATACTGAAGCTGGGTGGCCTAAATTTGATTCCAGGCTCCACCACCCTCTAGCTATGGGATCTGAACTTAAATTGTCTGTGACCTCGTCTGTATGCTAGGAACATAGTTAGCACCCAGGTCCAAAGATGGCTGGGGCCATGAGCAAGAAACCTGTGTGGCAGGCTGAGCCTGCAGTAAATACAGCTGTTGGGACCATCATGGGTCCTCTGGGTCCTCACACCTGGCCACCTGCATTTTCTGGATGCTGTGGTAGTATCCAAACACAGTAGTCCACAGGGTCCTGCAGGCTGGCCTTCCAGCAAGTCATAATGTTGGCTCAATCTCTCCATCTCAATCTCCCCCAATGAGAAATGGGATTGATAACACCTGTTAGGGCTACCGCAGGACTCAAAAGAAGCCACACAGCAAAGTTCCTTGCAGAGCTGGTGAGATGACCTTGCATTTGCTGTCGAGCCTGACTACCTTAGCTCCTTCCCCAGGATAGAAAGAGAGAATTAATACCCACAAGTTGTCCTCTGACTCCCACATGCACACTATGACATGTGTGTGCACACA |
| 11a | ACCCTGCTTCGAGACGGCAAACGAGAAAGCATTGTCAGCACCCTCTTCATCTCCCCAGGAGACGTGGAAAATGGACAGAGTATTGTGTGCCGAGCCACCAACAAAGCCATCCCCGGAGGAAAAGAGACCTCTGTCACCATAGACATCCAGC |
| 11b | GTGAGTACCAACTGACCCTCTCTGGCCTGGCCAGGCCTAGGGTTGGGACCAGCCCATGTGGTTGTGTGTGTGTGTGTGTGTGTGTGTGTGTGTGTGTGTGTATGCAGATTCATTGCAGATTCATACAACCTGTGCATGTGCACAGAGGCCAGAAGAAGTTGTTGGATGTCCTCCTCGATCAACTCTGCCTACTCTTTAGAGGCAACCTCTCTCACTGAACCTGGGTCATATGTTTTCTCTGCTAGGCTAAAGGCCATTAAGTTCAACTGTAAATGTAACTTTTATTTGCCTGGGTGATTATTGTCTCCAAGGCTTACTTCCTCAGTCTGCTATCCTAGGCCTAGTTCTGGAAGCTTCTAGTCTCTGTACAATCTTATCTAGGCCTAGAATGTTTTCAGCCTCTGAGACTTCCTGCTGAATAAGCTCACCCTTTCTAGTTCTTTTGACTGGCTGATTCAACTCAGTTGTTCTGGATCAAACTCCTCTCCAAACTGACTGATTCAATTACACTTTACTTTCAAATTCTTCTGAATCATTCTGCTTGTTCTGAAACTAACTCAAGCAATCTGTTCTAATCTGCTAGCTCATTCTCATTCTCGGGCTTGTTCTGTCTTCATCCACGGCTAGCTTGTTCTCCCTCTGCAGCCTGTCTCTGTACAACTAATCCCAGTAAAACTGCCTCCTTCCTCTCTGATGCTCTCTCTTCAAGTAGCTGCTCTTTCTTCTCCTGAGGGTTAGGCACCTCCTATTCTGTCAAATCTTTCTCTGATTCATCACTTTGTCTGCCACTCAATTAGACATTCTGTTCAAACATGGGTACTTCTTTCTACAAACTAACTTTATCTTCATCGTTTGGGATTAAAGGTGTATATAAGGGCACATGTGTATTCCAGCCAGAGGGGTTAAAGGTGTGTGCTAAATGTTGAGCCATACTACAACTAGGAACAGTTTTTTTTTTTTCTGTAAATGACACAATCTCAGGGTTCACAGTGTGATCAAATATCCTGCAAAACCCCAGCAGTTCTCCTGCCTATGTTCTCCTTGAAGCTGGAGTTATAGTTGTACACCAGGTGCCAGCCTGTTAAGTGGGGGCCAGTATTCAAAATCTCATCCTCATGTTTACGCTGCAAGTGCTTTTAACTACTAAGCCATCTCTCCAGTTCCATAATGCTGATGAATTTAAATACTTTCTAACAGCCCTGGCTCAGTTAGTGCTTCTGGGTTCTGTGTTGTATACACAGCTCTTCGGGGCTAAAGAGTGCACGGGAACTGTGTCTTGGGGTTCTCAGAGCCAGTTTTGACAGCCAGAGGACAGAAGTTTTCTCTGAGCAAATCCTAACTGACACCCTTTAAAATGCAGAAGGAAGAAGAGATGTTTGTTAAGTACTGGGTGAGGCTTAAACACCCAGGCAAACAACTCTCCTCTCCTCTCCTCTCCTCCACTCTCCTCTCCTCTCCTCTCTCTGTATTGCCATTTGTTGTTAACAAACCCCTTCTCCCATGCCAAGCTTGATCCTCAGTGAGAGTGAGAACAGCAGAATCCTTACCAAGCCTAGGGCTGGGGAGATAAAGAGACTGGAGAGCCAGACCATGGTGGCACACACCTTTAATCCCAGCACTCAGGAGGCAGAGGCAGGAGGATCTCTGTGAGTCTAAGGCCAGCCTGGTCTATAGAATGAGTTTCTAGGACAGCCATGTCTACAGAGAGAAACCCTGTATCAAAAACAAAAACAAAAAGAGACTGGAGAGAAGCACCTCGGCCATGTTCCTCCATCCCTGCCAGGATCCCACCCTGGGTCTTAGCACCATCCAGCAAGCAGATGTGATGAGAGCAAAGCCCAGCTTTGGAAAGTAGGTAAGATGCTAGTTCGGTATGAGGTTCAACATTCCGAACAGATAGATGCATTGCCATGAACTGGGTGGCTCCAGACTTGTCCTTTCACTCTGATAATTCGAATCTCTGGTCTGATTTGCTGATGCTATCCAAGATCTCTTCCTATTCTAGGAGCAATCAAAGCTCTCTCTCAAACACAAGCCAGATCTGGGGGGAATGGGGGTGGAAGACAATAAGTGTCTAAGTCCCTTGCTTCCTGGCTGCCATTTGTGGCTGTGGCACTTCTTAGGCAGGAGGCTATGACAGCTACAGAGTCCCACATAAACATAGTTGGTTCCCCCGAGATGGAAGCCAGGACCTTGACAGAGAAAGATGAAGGAGAAAGAACATCTCTGCCCAAGACTGATATCTGTTGCCCCAAGAGAACCTAGGGACAAGAATGCTATCATGAGAGTATCCCACATACATCCAAATGCATACCCCCTCCAGATGCACTCCACACAAAGGACTGGTTGACTGGCTACATGGTTAACAGGGCTATACCTGAAAGGACCTGGGTATGAAGCTAGCATGAGGCTTAGACCCAGGTTTCAGATTTCCCAGTTGGAATCTTTCCAAGGCAGTACAGAATAGTGGTTGTGGTCCAACATTCTCTCTAGTTTGAATTCTGGTTTTGCCTTTTACTGATTAGCCTCATTAGTAAATTGATTTGAGCCTTAGTTTCCTCAAGGCGATCATGTGTACCTCTTAAGGTCACAGCTAGAATTAAATGACATCATTCATGTCTATTCTTTGCACCATGCATGACATATTTTGATTAAAGTATATATTATGGTTGAAACCACGGTGGTGAGTGTGTGGTGACCCTTCCCCAGATGTCATCACATCTGTATCCATTTGATCCAACACTCCATCTCCCCTAGCTGGGAGGTGTGTTTATGTATACCTAACACGTAAGGTCCAACCCTTCTCCATGCCTTCCTCCTCCTCCTCCTCTTCTTCCTCTTCCTCCTCCTCCTCCACCTCTTCCTCCTCCTCTTCCTTTCTTCTCAAATTCACTTGCTCATTCAGTAAGCATTCAGCTGTTTGCAGTGGATACCACAGGCTGGATCAGAGCCGCCTTTGCAAACCTGCTCATTTTCTCAGTCAACCACAAAGTCTCCAAACTCAGCAAAAGCGCATTTGTTGTTTGTTTCCCTTGGTCTTTATATGTTACCCAACGGAAGACAAAGCCCCCAGATTGAGTAATGGGAGAGAAAGCGCAATCCCCCTCCCCTGACACACACACACACACACACACACACACACACACACACACACACACAAAGCCTCACCACATCTGGACTCTGTGTCCAGCTGCCAAGTTTCAAATGGATGTGCTATAAAATCACTGAGTCATGAAGTTTCTCTCTGCACCCTCCACACTTCTCCTGAAAATGCACATGTGCGCGCACACACACACACACAGGTTGCTGATGGAAACACTAACAGACGCTCAGCTGTAACATCTGTTCAATGTGATCTCAGTTCCACCCTCCTGCAAGTTATGCATCCGACATCTCAAGTGGGATTAAAATCATGTTTAATAGTTGGGTATGACATAAACTATTTATACCTCTGGAACTTGGCAGACTCCTAGAAACTCATATCCTGAGATGTGGCATCCCAGAGCCTGGGGATAGAGATGCAAATGAAACTGACTGCAGTGGACCCCTCTGGGTCTATTCTCCTTAGGCCCTAAGGACCAAGGGTGTTACAAGGGAAGGAGACGCTGATAACAGGGTATACAGGGCATCCAGCAAATGAAGACCCTCGAAGGGTCCCCCAGTAGGTATGTGTTCCTCCTTCTTGCTTCCCAAGAAATCTCACTCCATAAAGAGCACCAAGAAAAGGTTGAGCCTTTGGAAATTTCTCCCTGAGCTCTGTAGTGGGAATTTTGAACTGACACCATCATATCAAGCAAGAGTTGAACTGAGATTACCTATCACCCACCAGGAATGGAGTTTACAGTGTGAGGCTAGCAATGCACAGGGGTCCCAATGGCAATTAGCAAACTCCTCTGGACAAGCTCCTTTACCAGAGAGGATGCTCAGCTTGGCCAGCCTGCCCTGCACAGGACAGTGAGATTGTCAGCCTTTCCTGAGAAGCTGTGGGAGCCACGGGGAAGATTCTGTAGGACTTGCTGGGAGAGTTGCTGGGAGTCTAGGAAGTGAGAAGAGAATCCTGGTGATCCCTTGCCACAGATACCTTAGTAAGCCCTCGAATCTCACTTAAACGGATTCCAATGGTGAAGACCAGATATACCCTGAAAATTGACGACACCCCTAAACAGCAGGAAGTAGCTTAAGAGACACAACACCCTTATTCCCTCTTCACCATTGGTTTCCCCTCTCCTTTTCTCCCTTTCTTTTTATAATAACAAGAAGGGAGGAATGTTAGCATCAGGACACTCCAAGCCCAGGCCTAACTATGTGATCCTTGACCTGCATTGCCAGGACAATGACCCTCAATCCCACAATCCACTTGGCTCAGTTCACACCCTCACTGGTGTGACATCATTTTCCGTATAAATTAGGGAATCACCCCCTTCTCGCGCTCTCTCTTTCTTCTCTTTCTTCTCTCCCCCCTCTTTCTCTCTTTTTCCTCTTCTGTGCCCTTCTACCTGCTTCACCGCCCCTCTATCTCTGCACAATAAACCTCTCCCATGTGGAACCACCTTG |
| 12 | ATCCACCGCTTGTCAACTTGTCCGTGGAACCACAGCCGGTATTGGAGGACAACATCGTCACGTTCCACTGCTCTGCAAAGGCCAACCCAGCTGTCACCCAGTACAG |
| 13a | GTGGGCCAAACGGGGTCACATCATCAAGGAGGCATCTGGGGAGCTGTATAGGACCACGGTGGACTACACATACTTCTCAGAGCCTGTATCCTGTGAAGTAACCAATGCCCTGGGCAGCACCAACCTCAGCCGCACAGTGGATGTATACT |
| 13b | GTGAGTGTGGGGGCTCAGGGAACCCTGGGATCAGGCAGTGGGTGGTGTGTGGCTGAGACTATAGCTAGACTCATCACACAAACACAGTGTACATGTTGCGTGTTACAGCATCTCCTAGTACCACTCCTGTCTAGATCCTTGACTCCTCCCTTTGAGGCACCATCTCATAGTGCAGTGACCAGTTACTGAGGACTGGGAGGGCTGACCAGCAGGAATCAAGAAACAGCACTACTGTTCTGGTGCCATGGATCCCAATCTTCTCCCAAGGACCCTACCTCCTAATGCATCCACAATGGGGGTTTCAACATGTAAATCTGAGGGGGTCACATGTACAGTCCATAAGTATATCTTATTTCCCTATTATCAAAATGAGGGAGATGTACTCTCAAGGGCATGCAGGGAGTAAGTATCAGGGTGAGGACTGAAGCCAGACAATAACTAAATCCATCACCGATACACACTCCACTGTCCCACTGAGGGGACACATGGACAGCTTCCTTTCCTCACCAGTGGCCCAACAGATGAAGCCAACCTACTTTTAAGGACTAGCCAGATGGATGT |
| 14 | TCGGTCCTCGAATGACCTCAGAGCCTCAGTCACTGCTGGTAGATCTGGGCTCCGATGCTGTCTTCAGCTGTGCGTGGATCGGCAACCCGTCTCTGACCATCGTGTGGATGAAACGAGGTTCTGGTGTG |
| 15 | GTCCTGAGCAATGAAAAGACCCTAACCCTCAAATCTGTCCGCCAAGAGGATGCTGGGAAGTACGTGTGCCGGGCTGTGGTGCCCCGGGTAGGAGCTGGGGAGAGAGAGGTGACCTTGACTGTCAATG |
| 16 | GACCCCCCATCATCTCCAGCACACAGACCCAGCACGCCCTCCACGGAGAGAAGGGCCAGATCAAATGCTTCATCCGGAGCACACCACCGCCTGACCGAATT |
| 17a | GCCTGGTCCTGGAAGGAGAATGTGCTGGAGTCAGGGACATCAGGGCGCTACACAGTGGAGACGGTGAACACGGAGGAGGGAGTCATCTCCACATTGACCATTAGCAACATTGTGCGTGCTGACTTCCAGACCATATACAACTGTACAGCCTGGAACAGCTTTGGCTCTGACACAGAGATCATCCGACTCAAGGAACAAG |
| 17b | GTGAGGACTGCCAGGGTGCCCCGGGGACCAAGGATTGCCGGCCAGATCTAAGGACGGGCCTCACACTCTTCCTCACTGCAATGCAGCCTCACCTTCTGCTGCCAGGGGCACTGGCCCCTCCAAGGAAACAAAGATACTCCGAGTGATGCCCTCTGGGACTGCTCAGGAAAGGGTGACCACAGAGTGAGCTTGGCTGATGGCAGTGATAAACACCCAATGGTGTCAAACACCCTGGCTTCCACACACTCTGCCTTCCCCTGAGGGATCTGAGCCCGAATGCCCTTGCTAGGTTGCGCTTGGACATACATGCTGAGGAATTAGCCCAAACATGATAAAACAGAAGTCCCAAGAATGAGCCAATGCTAAAGCCAAAGAGTAGCGATGCTTATGTGAGGGAGTGGGGGCTGGGCTCAGACCACTTGCAACCCCCAGGAGACATCTGGACCCTAGAGGAGACTCTGATTAGTACTGGAGTACAGAGAAGCTGGATTTTGAGAGGTGGAGAAAGAGGAAGGAAGCTTACCTCTCTGTAATCCATCACTATGGCCCCATATGCCAACTGCCCTCACTAGTGACGCTAAGAACTTGGTCTCATGCTAGACATGGTAGCATGTGCTACTCTTAGTTACACAATAGACTGAGGTAGCAGGATCATTTGAGCCCACACATTCAAGAACAACCTGGGGAACATAGCAAGCCCTTTTCTCAGAACAAACAAGAGATCTCCAGTCTCCATTTAAAGTTCTCCAGCTCAGACTACCAAATGGAGAGTGCCAGAAAGATGACAGCCAGTCCAGAGAAGTTCACTCAGCACATAGTCAGTAGCACTAGGTCCTGGAACGGACTAAAGAGCTTCCCTCTGCCAGCTAGTTCCCAGCCACGGGGTTCAGGGGTGTCCTGGACCTCTGGACCAGAAGTTGGAAGCCCAGCAAGTGGTCCCATCCAGACCCCACCTCCACCCCCCACCCCTGCAAGGCTCAATAAATGAGGTGGCCGGTGATCACACCTACA |
| 18 | GTTCGGAAATGAAGTCGGGAGCCGGGCTGGAAGCAG |
| 19a | AGTCTGTACCAATGGCCGTCATCATCGGGGTGGCCGTAGGAGCTGGCGTGGCCTTCCTCGTCCTAATGGCAACCATTGTGGCCTTCTGCTGTGCCCGTTCCCAGAGAA |
| 19b | GTACGGGAGGGAGACCCGGGATCTCAGGGAGGGGGACAGAGAAAAAGGCCAGGCTTAGACTGCCCAGGAGAGCAA |
| 20a | ATCTCAAAGGTGTTGTATCAGCCAAAAATGATATTCGAGTGGAAATTGTGCACAAGGAGCCATCTTCTGGCCGGGAGGCTGAGGACCACACCACCATAAAGCAGCTGATG |
| 20b | GTAAGAGCACAGCCTATGCCCCACTCCATCCTGAGCACACAGACTTCCCGATGCTCTCCATACTGCTGACAGGTAGCACATCCAGGCAAACCCCTCCTGCCCCCAAAGTAAGCCGGGCATGCACTCAGCCAATGAGCATTTATTTGTGCCTCTGTGACGTGATGGGGGAGATTCAAATACAACTTTGATGCAGTTCTTGACCTGGATCCTGACCACTGAGTAAGGAAAGAACCTGTGCTTGAATTCACATGTGTGGCTGCGTACATGTGAATTCAATGTGATCCTCTTCCTGATCCTCTCTGCAAGAACTGGAATCCTCCCAACTGTCCAGGCAGACTCACATGGGGAGAGGCATGGTATAGATATTCGATGCATTTTTTCTCCTCTTCCCTCTTCACACACACACACACACACATCTGCCAGAAACCCTGGTTTCTTCTTGGGAACTGGATATCCACCAAAAGAAAATGCAAAACTTCTCCAGCAACACCAAACTGCCAACTCCTCTTCCTGATCCTCTCTGCAAGAACCCGAATCCTCCCAACTGTCCAGGCAGAAGTCTAGTCAACTATGCCGGAAGCTCACAGAACACTATATCGTGGTGGCATTATCTGGGAACAGTGACAAGAGGGCAAGGAATAAAGGACAAGGGCTTTTCTGAGCCCCAGTGACCCCATCTATGAAATGGACATGAACACAGTAATATACAGAGCCATCATCAGGATTATAGGGTAACAACCTGAGTACATACGGGGTACAGTAGAGTTTTCCCTCGACATATGTGCAGAGTTCTGTCCTATAATTCCCAGATACCCAAATCTGAATGTGTACAAGTATAAAATGCTATATAACATTTATACAGATGCTACCTGCTTCCTCTCATATTCATAAATCATCTCTACTTATGAAGTGTAATGTAAGTGCTGTGTAAACAATTACTGTACCATATTGCTTAAGGATTAATGGCGAGTAAAATATGCACGTTTGATACAGGC |
| 21 | ATGGACCGGGGTGAATTCCAACAAGACTCGGTGCTGAAACAGCTGGAGGTCCTCAAAGAAGAGGAGAAGGAGTTTCAGAACCTGAAG |
| 22 | GACCCCACCAACGGCTACTACAGCGTCAACACCTTCAAAGAACACCATTCAACTCCAACCATCTCCCTGTCCAGCTGCCAGCCAGACCTGCGTCCGACAGGCAAACAGCGTGTGCCCACAGGCATGTCCTTCACCAACATCTACAGCACCTTGAGCGGCCAGGGCCGCCTCTACGACTATGGACAGAGGTTTGTGCTGGGCATGGGCAGCTCTTCCATTGAGCTTTGTGAGCGGGAGTTTCAGAGGGGCTCCCTCAGCGACAGCAGCTCCTTCCTGGACACGCAGTGTGACAGCAGCGTCAGCAGCAGCGGCAAGCAAGATGGCTACGTGCAGTTTGACAAGGCCAGCAAGGCTTCTGCCTCCTCTTCCCACCATTCCCAGTCCTCTTCCCAGAACTCCGACCCCAGCCGACCCCTGCAGCGGCGGATGCAGACTCACGTCTGAGGACCACGCCCTGTGGTGGGGGATGGGCCAAGGAGGAGGACATGGTACATTCTCGTTCTCCAAGGATTGGGGCTACTTTGCAGAGGACCCTAGAACTGGCCACCTCCGGGGTGGTCTCCGAGCACCTCTGTAAACACCTTCCTTCAAAGCTCTGATCAAGCACAAATCTGGCTCCCAGGTGGGAAATGGAGAGTATGCAGCTGAGCGGATAGTGCTCAGGGCCTCTGTCTCTTGCTCTTCCCTAAAGGTCCCTCAACCACCTTGTCCTCCCATGGGCACTCGTGGCAGCTAGAACTTTGCTTTTATGAAACTGCCGTCCACTTTCCTAGCTCCTCTTGCTGCCCATAAGCCATCCCTGGTGTCTGTATTCCTTGCAGCCTTGAGGAACGGAGGACTTTTTCCCAGCACTGAGCTGCTCCGGAGACCCCAGCCTCCCCACTGTGCATAGCCTATACCGCAGAGGCTGGGGCCTGAGAAATGGCCCTGACCAAAGGAGCATCTGCCTGGGAGTCCGCCCCCACTTTGTTTGGTGTTTGTGTCTGTATTCTTGCAGTTCTGTTCTTGGACTTGATACCTCTGCGCTTGGTGGTGGGACTGGCCTATCAGAGTCTAGTGTCCTCAGAGCTGAGGAAGGGAAAGAGGGAAAATGTGAACTCCTGGAGAACAACTGGCCCAACACACCCTGTGCCAGGCTGTGCAGTTCAGAGCCCTCACCGTCATCCTCACCCCCTGCCCCGTGTTCTCCCTTCCTTCCCACAGCACAATCGAGCTAATCCGAGGAGTGTGAGAACTCCTCTTGTCAGGGTTTTTTGAACAGTTACTGAAGCGTGCTTCCTGGGAGATGTGGGTTTGAGGGGGTGCTGAAATCTAGGCTGGAGGATGAGACAGACTCTTTCAGCTGATGACCACAAGGAACAATGATCCATTCTCCAGTAGATAGGACTCTGTGTGCAAGAGGGACAGTTTTCTTCACCTCTTTCCCATCACTCCCCACTTAAGAATAAACGTTAGGGCCATTACCCCCAACAAA |

**Human Kirrel3 gene (NCBI Gene 84623)**

| exon |  |
| --- | --- |
| 1 | GCGCCGCCCCCGGACGCGAAGGCTTCCAGCAGGGGCGGCGGTCTCTCTCCTCTCCCCTCTTGCAGCGCACTCCGAGGTCTAGGGACTCTCCGCGCTTCTCCATCAGGTGGAGCCGTCGGCTCCTCGCCGCCTCCCTCCTCCTCCCTCCCCCGGAGCGAACCGCGCGCAGGCAGCCTTGCAGCGCCAGGAGGCTAACCGAGCCCCCGGAGGAGGTGAGGCCGCGGGCAGCCGGGCGGAGATGGCGGGGCCGCCGTCGAGGCTGGCGCCAGAGCCCGACCGGCTGTGGGTCCTGCAG |
| 2 | CTTCAGTGAAAGGAGTCCTTCTGTCACTCGTCACTAGCTCGCTCGCTCACTGTGGGAGGAGCCCGCCTGAGGAAGCCGTGTGCCTGGGATGCCAAGAGCCAGAGAATGGATCTTCTCCGAGTGGGGACATTGCTGACAATCCCGGCTTCCCGAGGCGGCTAAGAACAGGCAGTTTGTGTCGGCTGGCTGCAGATACCCAGAGGCACAAAGAGACCGAAGCCACCCGGAGGGACCCACGGACGGACAGATGGTAGGCGCGAACCCGAGAGGACCGGCGGAGGCTGAGCACCGAGAGCCGCCAAGGAAGAGAAACTAACCACAGCCAAGTTACCCCGCCGGCTTTCCTTCGCTGCGCTAAGGAATGAAACCCTTCCAGCTCGATCTGCTCTTCGTCTGCTTCTTCCTCTTCAGTCAAG |
| 3 | AGCTGGGCCTCCAGAAGAGAGGATGCTGTCTGGTGCTGGGCTACATGGCCAAGGACAAGTTTCGGAGAATGAATGAAG |
| 4 | GCCAAGTCTATTCCTTCAGCCAGCAGCCCCAGGACCAGGTGGTGGTGTCGGGACAGCCAGTGACGCTACTTTGCGCCATCCCCGAATACGATGGCTTCGTTCTGTGGATCAAGGACGGCTTGGCTCTGGGTGTGGGCAGGGACCTCTCAA |
| 5 | GTTACCCACAGTACCTGGTGGTAGGGAACCACCTGTCAGGGGAGCACCACCTGAAGATCCTGAGGGCAGAGCTGCAAGACGATGCGGTGTACGAGTGCCAGGCCATCCAGGCCGCCATCCGCTCCCGCCCCGCACGCCTCACAGTCCTGG |
| 6 | TGCCGCCTGATGACCCCGTCATCCTGGGGGGCCCTGTGATCAGCCTGCGTGCGGGGGACCCTCTCAACCTCACCTGCCACGCAGACAATGCCAAGCCTGCAGCCTCCATCATCTGGTTGCGAAAGGGAGAGGTCATCAATGGGGCCACCTACTCCAAG |
| 7a | TGACAGGATCTTCCTATGTTGCCCAGGCTGGTCACCAAGTCCTAGGCTCAAGCAATCCTCCCACCTCAGCCTCCCAAAGTGCTGGGATTACAGACATGAGCCTCCACACCCAGCAGACTCTCTTCTCTTCTTCCAAGGACACCAGTCATTGGGTTTAAGGCTCACTCTGAATCCA |
| 7b | GTATGACCTCATCTCAAGAGCCTTAACTAATTACTAATTGCTTAACTAAACTGCAAAGACCCTATTCCCAAATAAGGTCATATTCTGAGGTTCCTGGTAGACAAGAATTTGGAGAGGACACCATTCAACCTGCTGTAGGAGACTAGTACTTATTTCAGGACCAACTTGAAAT |
| 8 | GCCTGAGACTGTCACTCAAGCACCAGAGGCAAAAAGATG |
| 9 | ACCCTGCTTCGGGACGGCAAGCGGGAGAGCATCGTCAGCACCCTCTTCATCTCCCCTGGTGACGTGGAGAATGGCCAGAGCATCGTGTGTCGTGCCACCAACAAAGCCATCCCCGGAGGAAAGGAGACGTCGGTCACCATTGACATCCAGC |
| 10 | ACCCTCCACTGGTCAACCTCTCGGTGGAGCCACAGCCAGTGCTGGAGGACAACGTCGTCACTTTCCACTGCTCTGCAAAGGCCAACCCAGCTGTCACCCAGTACAG |
| 11 | GTGGGCCAAGCGGGGCCAGATCATCAAGGAGGCATCTGGAGAGGTGTACAGGACCACAGTGGACTACACGTACTTCTCAGAGCCCGTCTCCTGTGAGGTGACCAACGCCCTGGGCAGCACCAACCTCAGCCGCACGGTTGACGTCTACT |
| 12 | TTGGGCCCCGGATGACCACAGAACCCCAATCCTTGCTCGTGGATCTGGGCTCTGATGCCATCTTCAGCTGCGCCTGGACCGGCAACCCATCCCTGACCATCGTCTGGATGAAGCGGGGCTCCGGAGTG |
| 13 | GTCCTGAGCAATGAGAAGACCCTGACCCTCAAATCCGTGCGCCAGGAGGACGCGGGCAAGTACGTGTGCCGGGCTGTGGTGCCCCGTGTGGGAGCCGGGGAGAGAGAGGTGACCCTGACCGTCAATG |
| 14 | GACCCCCCATCATCTCCAGCACCCAGACCCAGCACGCCCTCCACGGCGAGAAGGGCCAGATCAAGTGCTTCATCCGGAGCACGCCGCCGCCGGACCGCATC |
| 15 | GCCTGGTCCTGGAAGGAGAACGTTCTGGAGTCGGGCACATCGGGGCGCTATACGGTGGAGACCATCAGCACCGAGGAGGGCGTCATCTCCACCCTGACCATCAGCAACATCGTGCGGGCCGACTTCCAGACCATCTACAACTGCACGGCCTGGAACAGCTTCGGCTCCGACACTGAGATCATCCGGCTCAAGGAGCAAG |
| 16 | GTTCGGAAATGAAGTCGGGAGCCGGGCTGGAAGCAG |
| 17a | GAGGTGGCAGTGGAGAAGCTGTAACCCTGCTCCCTGGACCCTGAGACTTGGACACCTGAGGGCCACAACTGATGCCCAGCAGACCTGCGGGGAAGCTACTTAGACCTGCAAACACTAAGAGCCGGATGTCCTCTATATTCACCCGAGAAAGGGAAGGGAAGGAGAGAACATACGGAACAAACAAGGGCAGTGACCATAGTCATTTTTCAAAAAAAGCCAGGGCCTATGACTCACTGGCCGCTGTGTGATAGAGCATTCCTGTTTAATGCCAGAGAGAGCTCCATCGTGCCAGCCCCACCCCTCATCTCCGGTCTTCCTGTCCCCTGGCCCTCCCTTCTCCGTGTCTGCCTGCCTCGCTCTCCTTCCTCTGCCCTTTACCTCAGTTCCCCTGCCCTCTCCTCCACCCACCAAGCCCCTGTGTCCTCCACCCTGGTCTTTGCAGTCCCAGCTAGAGCTGACCCCAGTTGGACTCGGCCTGTGCCTGCCCAGGCCCCACTGAGGCATTTCCCAGGAGGGCCCCATGTGGCAGGGGCTGCCCCCGCAGGAACGGGGTGAGATCATGGGGGGATAGTAGGCCATTCCCCATCCGTCATCAGCTGTGCCCGCTTCTCTCCCACAGA |
| 17b | AGTCTGTGCCGATGGCCGTCATCATTGGGGTGGCCGTAGGAGCTGGTGTGGCCTTCCTCGTCCTTATGGCAACCATCGTGGCGTTCTGCTGTGCCCGTTCCCAGAGAA |
| 17c | GTACGGGAGGGAGATCCGGGATCTCAGGGAGGGGGACAGAGAAAAAGGCCAGGCTTAGGCTGCCCCGGAGAGCAA |
| 17d | GTAAGCAGGAGTGCAATGAACAGGGGTCCTAACAGTGCTGTGAGCTCCTGGGGCAGGGAGTGGGTCTGATGCATCGGTGTATGTGAGCCTGGGCAACATGGCGCCTGGCAGAGTGGGCGCTAGGCTGAGGTTGACCTGGACTAGACTGAACTTCATCTGCAGGGCAGCCAGCATTTTGGATTGAACACATAGCTCTTTCAGTCAGGAACTGTACAGAAAGATAGGGGGAAAAGCGGTTTGTGGTTTGATCCTTGCTCTACAAGAGCTGTTAGTCTAGAGAGACCCCATCTCTACAACAAAATAAAAATAAAGAGCTGCTAGTCTCACCAGAAAAGCAGGTCACTCACACAGCTGTGGGGGAGTGGGTGGGGAAGCAATAAAGGAATTGCTTTGAGAAAACTTTT |
| 18 | ATCTCAAAGGTGTTGTGTCAGCCAAAAATGATATCCGAGTGGAAATTGTCCACAAGGAACCAGCCTCTGGTCGGGAGGGTGAGGAGCACTCCACCATCAAGCAGCTGATG |
| 19a | GCAACTGGCCGGCATTTTACAATAAACGTTCAGTCAATGGAATTGAATCAGCCATGGGAGATCTCTGGTGATGCCCTCGCCCAAGCGAGG |
| 19b | AAAGTATGGCCTCGGATACCTGCCCCTCGCCTCCCCTAACTGCCCCTCACCTCACCTACTCACCCACCCCCCTCAACTGCTTCCCTGTCTCCTAGGATGGGGCTCCAGGAACTCATCTTGACTCTTTCACATGGTCCAG |
| 19c | GCAGTGAGGGGTGGTGGGGGTTATACAGGGCAATCTTTTGAGTTTCTGATCTCATCTTTTCCTTGGGGGAAGTGGAGGTTTAGCCACCTCCTATCTACTGAGCAGCCTTTTTTGGGTTGC |
| 20 | ATGGACCGGGGTGAATTCCAGCAAGACTCAGTCCTGAAACAGCTGGAGGTCCTCAAAGAAGAGGAGAAAGAGTTTCAGAACCTGAAG |
| 21 | GACCCCACCAATGGCTACTACAGCGTCAACACCTTCAAAGAGCACCACTCAACCCCGACCATCTCCCTCTCCAGCTGCCAGCCCGACCTGCGTCCTGCGGGCAAGCAGCGTGTGCCCACAGGCATGTCCTTCACCAACATCTACAGCACCCTGAGCGGCCAGGGCCGCCTCTACGACTACGGGCAGCGGTTTGTGCTGGGCATGGGCAGCTCGTCCATCGAGCTTTGTGAGCGGGAGTTCCAGAGAGGCTCCCTCAGCGACAGCAGCTCCTTCCTGGACACGCAGTGTGACAGCAGCGTCAGCAGCAGCGGCAAGCAGGATGGCTATGTGCAGTTCGACAAGGCCAGCAAGGCTTCTGCTTCCTCCTCCCACCACTCCCAGTCCTCGTCCCAGAACTCTGACCCCAGTCGACCCCTGCAGCGGCGGATGCAGACTCACGTCTAAGGATCACACACCGCGGGTGGGGACGGGCCAGGGAAGAGGTCAGGGCACGTTCTGGTTGTCCAGGGACGAGGGGTACTTTGCAGAGGACACCAGAATTGGCCACTTCCAGGACAGCCTCCCAGCGCCTCTGCCACTGCCTTCCTTCGAAGCTCTGATCAAGCACAAATCTGGGTCCCCAGGTGCTGTGTGCCAGAGGTGGGCGGGTGGGGAGACAGACAGAGGCTGCGGCTGAGTGCGCTGTGCTTAGTGCTGGACACCCGTGTCCCCGGCCCTTTCCTGGAGGCCCCTCTACCACCTGCTCTGCCCACAGGCACAAGTGGCAGCTATAACTCTGCTTTCATGAAACTGCGGTCCACTCTCTGGTCTCTCTGTGGGCTCTACCCCTCGCTGACCAGAAGCTCTACCTACCCCTGTGCCTGTGCTCCCATACAGCCCTGGGGAGAAGGGGATGACGTCTTCCCAGCACTGAGCTGCCCCAGAAACCCCGGCTCCCCACTGCTGCTCATAGCCCATACCCTGGAGGCTGACAAGCCAGAAATGGCCTTGGCTAAAGGAGCCTCTCTCTCACCAGGCTGGCCGGGAGCCCACCCCCAATTTGTTTGGTGTTTTGTGTCCATACTCTTGCAGTTCTGTCCTTGGACTTGATGCCGCTGAACTCTGCGGTGGGACCGGTCCGGTCAGAGCCTGGTGTACTGGGGGGAGGGAGGGAGGAGGGAGCCTGTGCTGACGGAGCACCTCGCCGGGTGTGCCCCTCCTGGGCTGTGTGACCCCAGCCTCCCCACCCACCTCCTGCTTTGTGTACTCCTCCCCTCCCCCTCAGCACAATCGGAGTTCATATAAGAAGTGCGGGAGCTTCTCTGGTCAGGGTTCTCTGAACACTTATGGAGAGAGTGCTTCCTGGGAAGTGTGGCGTTTGAAGGGGCTGGAGGGCAGGTCTTTAAGATGGCGAGACTGCCCTTCTCAGCTGATAAACACAAGAACGGCGATCCTGTCTTCAGTAAGGCTCCACGAGAAGAGAGGAAGTATATCTACACCTCAACCCTCCTAGTCACCACCTGAAATAAATGTTAGGGACACTACTCCA |

**Mouse SRA Kirrel3 transcripts**

**brain region SRA file Kirrel3 transcripts total transcripts**

hippocampus SRX18486306 123 2,961,269

hippocampus SRX18486305 186 3,124,583

hippocampus SRX18486105 147 2,100,786

hippocampus SRX18486104 124 1,874,595

hippocampus SRX18486098 95 3,038,371

hippocampus SRX18486306 123 2,961,269

hippocampus SRX18486305 186 3,124,583

hippocampus SRX18486105 147 2,100,786

hippocampus SRX18486104 124 1,874,595

hippocampus SRX18486098 95 3,038,371

hippocampus SRX18486097 100 2,991,115

other SRX1631051 22 16,225

other SRX1631053 1 40,643

other SRX4218970 266 5,219,223

other SRX4515335 94 1,309,970

other SRX5989879 2 169,679

other SRX5989880 10 242,895

other SRX5989882 3 93,712

other SRX9178674 193 10,730,073

other SRX9178675 187 9,508,271

other SRX9178677 9 3,371,331

**Human SRA Kirrel3 transcripts**

**brain region SRA file Kirrel3 transcripts total transcripts**

hippocampus SRX18485725 25 1,421,315

hippocampus SRX18485724 33 1,440,224

hippocampus SRX9141829 64 3,176,068

hippocampus SRX9141830 47 3,136,724

other SRX10976497 1 649,166

other SRX11141673 17 1,121,497

other SRX11141674 25 1,487,748

other SRX11141675 314 20,213,461

other SRX11141676 21 4,826,345

other SRX1743232 3 1,179,457

other SRX19335730 44 2,888,177

other SRX8068351 4 6,363,354

other SRX8068354 2 28,141,773

other SRX8068356 319 184,339,749

other SRX9014757 4 355,152

other SRX9141821 2 12,994,243

other SRX9141822 13 16,258,243

other SRX9141824 226 21,071,378

other SRX9141825 201 9,584,363

**Extended Data, Table 2-1: Mouse and Human Kirrel3 exon sequences and data bases analyzed.** Sequences of the 22 mouse and 21 human Kirrel3 exons. Segments that extend an exon are labeled with the letter ’b’, the respective non-extended exons are labeled with ‘a’. NCBI Sequence Read Archive (SRA) data used to search for mouse and human Kirrel3 transcripts.
