# Supplementary figures and images for: Modular Splicing Is Linked to Evolution in the Synapse-Specificity Molecule Kirrel3
Source: eNeuro. 2023 Dec 4;10(12):ENEURO.0253-23.2023. doi: 10.1523/ENEURO.0253-23.2023 (PMC10698715; doi:10.1523/ENEURO.0253-23.2023)

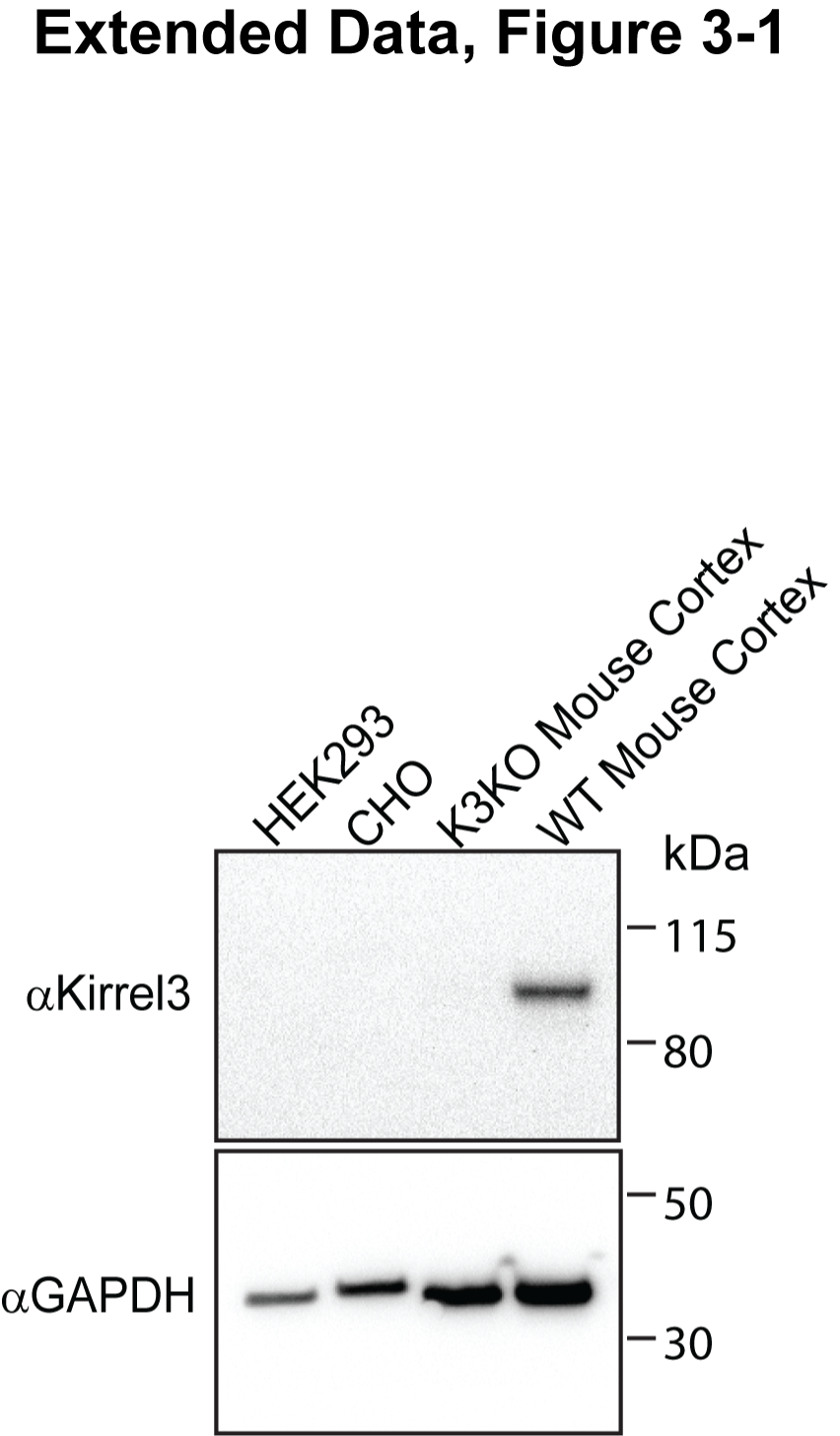

Supplement: Extended Data Figure 3-1 — HEK293 and CHO cells do not express endogenous Kirrel3. Western blotting showing that HEK293 and CHO cell lines used here do not endogenously express detectable levels of Kirrel3 protein. Kirrel3 WT and KO mouse cortex samples were used as positive and negative controls. Download Figure 3-1, TIF file. [file enu-eN-NWR-0253-23-s02.tif]
