# Supplementary material for: Modular Splicing Is Linked to Evolution in the Synapse-Specificity Molecule Kirrel3
Source: eNeuro. 2023 Dec 4;10(12):ENEURO.0253-23.2023. doi: 10.1523/ENEURO.0253-23.2023 (PMC10698715; doi:10.1523/ENEURO.0253-23.2023)
Supplement: Extended Data Table 3-1 — Data analyses. Details of statistical analyses used in Figure 3. Download Table 3-1, DOCX file. [file enu-eN-NWR-0253-23-s06.docx]

**Stats Table Referring to Figure 3**

| Figure | Data structure/Normality | Type of test | results |
| --- | --- | --- | --- |
| Aggregation assay (fig 3B) | ND, sample size too small to test | Ordinary one-way ANOVA | P=0.003 |
| Junction assay (fig 3D) | normal distribution | Nested one-way ANOVA | P=0.0005 |

**Extended Data, Table 3-1: Data Analyses.** Details of statistical analyses used in Figure 3.
