# Supplementary material for: Modular Splicing Is Linked to Evolution in the Synapse-Specificity Molecule Kirrel3
Source: eNeuro. 2023 Dec 4;10(12):ENEURO.0253-23.2023. doi: 10.1523/ENEURO.0253-23.2023 (PMC10698715; doi:10.1523/ENEURO.0253-23.2023)
Supplement: Extended Data Table 4-1 — Fluorescent in situ hybridization (FISH) probes. Sequences of probes used in FISH experiments. Download Table 4-1, DOCX file. [file enu-eN-NWR-0253-23-s05.docx]

**Extended Data, Table 4-1**

**Mouse Kirrel3 exon 20b probes**

1 AAGAGCACAGCCTATGCCCCACTCCATCCTGAGCACACAGACTTCCCGATGC

2 AGTAGAGTTTTCCCTCGACATATGTGCAGAGTTCTGTCCTATAATTCCCAGA

3 TACCTGCTTCCTCTCATATTCATAAATCATCTCTACTTATGAAGTGTAATGT

4 TCCATACTGCTGACAGGTAGCACATCCAGGCAAACCCCTCCTGCCCCCAAAG

5 AGCCGGGCATGCACTCAGCCAATGAGCATTTATTTGTGCCTCTGTGACGTGA

6 AGTAAGGAAAGAACCTGTGCTTGAATTCACATGTGTGGCTGCGTACATGTGA

7 TCAATGTGATCCTCTTCCTGATCCTCTCTGCAAGAACTGGAATCCTCCCAAC

8 CTCCTCTTCCCTCTTCACACACACACACACACACATCTGCCAGAAACCCTGG

9 AACTGTCCAGGCAGAAGTCTAGTCAACTATGCCGGAAGCTCACAGAACACTA

10 GGCTTTTCTGAGCCCCAGTGACCCCATCTATGAAATGGACATGAACACAGTA

11 ATACAGAGCCATCATCAGGATTATAGGGTAACAACCTGAGTACATACGGGGT

12 GGGGAGATTCAAATACAACTTTGATGCAGTTCTTGACCTGGATCCTGACCAC

13 TCCAGGCAGACTCACATGGGGAGAGGCATGGTATAGATATTCGATGCATTTT

14 ACCAAACTGCCAACTCCTCTTCCTGATCCTCTCTGCAAGAACCCGAATCCTC

15 TCGTGGTGGCATTATCTGGGAACAGTGACAAGAGGGCAAGGAATAAAGGACA

16 CCCAAATCTGAATGTGTACAAGTATAAAATGCTATATAACATTTATACAGAT

17 GTGCTGTGTAAACAATTACTGTACCATATTGCTTAAGGATTAATGGCGAGTA

**Mouse Kirrel3 exon 22 probes**

1 GACCCCACCAACGGCTACTACAGCGTCAACACCTTCAAAGAACACCATTCAA

2 CCAACCATCTCCCTGTCCAGCTGCCAGCCAGACCTGCGTCCGACAGGCAAAC

3 CGTGTGCCCACAGGCATGTCCTTCACCAACATCTACAGCACCTTGAGCGGCC

4 GGCCGCCTCTACGACTATGGACAGAGGTTTGTGCTGGGCATGGGCAGCTCTT

5 ATTGAGCTTTGTGAGCGGGAGTTTCAGAGGGGCTCCCTCAGCGACAGCAGCT

6 TTCCTGGACACGCAGTGTGACAGCAGCGTCAGCAGCAGCGGCAAGCAAGATG

7 TACGTGCAGTTTGACAAGGCCAGCAAGGCTTCTGCCTCCTCTTCCCACCATT

8 CAGTCCTCTTCCCAGAACTCCGACCCCAGCCGACCCCTGCAGCGGCGGATGC

**Mouse GAD1 probes**

1 ATggCATCTTCCACTCCTTCgCCTgCAACCTCCTCgAACgCgggAgCggATC

2 AATACTACCAACCTgCgCCCTACAACgTATgATACTTggTgTggCgTAgCCC

3 ggATgCACCAgAAAACTgggCCTgAAgATCTgTggCTTCTTACAAAggACCA

4 AgCCTggAAgAgAAgAgTCgTCTTgTgAgCgCCTTCAgggAgAggCAgTCCT

5 AAgAACCTgCTTTCCTgTgAAAACAgTgACCAgggTgCCCgCTTCCggCgCA

6 gAgACCgACTTCTCCAACCTgTTTgCTCAAgATCTgCTTCCAgCTAAgAACg

7 gAggAgCAAACTgCgCAgTTCTTgCTggAAgTggTAgACATACTCCTCAACT

8 gTCCgCAAgACATTTgATCgCTCCACCAAggTTCTggATTTCCACCACCCAC

9 CAgTTgCTggAAggCATggAAggCTTTAATTTggAgCTgTCTgACCACCCCg

10 TCTCTggAgCAgATCCTggTTgACTgTAgAgACACCCTgAAgTACggggTTC

11 ACAggTCACCCTCgATTTTTCAACCAgCTCTCTACTggTTTggATATCATTg

12 ATCgTTggATggTCAAATAAAgATggTgATgggATATTTTCTCCTgggggAg

13 ATATCCAATATgTACAgCATCATggCTgCTCgTTACAAgTACTTCCCAgAAg

14 AAgACAAAAggCATggCggCTgTgCCCAAACTggTCCTCTTCACCTCAgAAC

15 AgTCACTATTCCATAAAgAAAgCCggggCTgCgCTTggCTTTggAACCgACA

16 gTgATTTTgATAAAgTgCAATgAAAgggggAAgATAATTCCggCTgATTTAg

17 gCAAAAATTCTTgATgCCAAACAAAAgggCTATgTTCCCCTTTATgTCAATg

18 ACCgCAggCACgACTgTTTACggAgCATTCgATCCAATCCAggAAATTgCgg

19 CTgCTCATgTCCCggAAgCACCgCCACAAACTCAgCggCATAgAAAgggCCA

20 ATTCTggTCAAggAAAAgggTATACTCCAAggATgCAACCAgATgTgTgCAg

**Extended Data, Table 4-1: Fluorescent in situ hybridization (FISH) probes.** Sequences of probes used in FISH experiments.
